# Correction to ‘Probabilistic cell/domain-type assignment of spatial transcriptomics data with SpatialAnno’

**DOI:** 10.1093/nar/gkad1229

**Published:** 2023-12-18

*Nucleic Acids Research*, Volume 51, Issue 22, 11 December 2023, Page e115, https://doi.org/10.1093/nar/gkad1023

In the originally published version of this manuscript, the data availability was incorrectly stated. The statement should read, “This study made use of publicly available datasets. These include the mouse OB dataset (https://www.spatialresearch.org/), DLPFC dataset on the 10x Visium platform are accessible at (https://github.com/LieberInstitute/spatialLIBD), seqFISH dataset (https://doi.org/10.18129/B9.bioc.MouseGastrulationData), and mouse hippocampus Slide-seq and Slide-seqV2 datasets (https://singlecell.broadinstitute.org/single_cell/study/SCP948/robust-decomposition-of-cell-type-mixtures-in-spatial-transcriptomics). The SpatialAnno software and source code have been deposited at https://github.com/Shufeyangyi2015310117/SpatialAnno. The code underlying this article is available in Zenodo at https://doi.org/10.5281/zenodo.7414189.”

This error has been corrected online.

