# Supplementary material for: Probabilistic cell/domain-type assignment of spatial transcriptomics data with SpatialAnno
Source: Nucleic Acids Res. 2023 Nov 6;51(22):e115. doi: 10.1093/nar/gkad1023 (PMC10711557; doi:10.1093/nar/gkad1023)
Supplement: gkad1023_Supplemental_Files [file gkad1023_supplemental_files.zip › 4 Supplementary Notes.pdf]

Supplementary Notes

Probabilistic cell/domain-type assignment of spatial transcriptomics data with SpatialAnno

Xingjie Shi[1,†,*], Yi Yang[2,†], Xiaohui Ma[3], Yong Zhou[1], Zhenxing Guo[4], Chaolong Wang[5], Jin Liu[4,*]

[1]KLATASDS-MOE, Academy of Statistics and Interdisciplinary Sciences, School of Statistics, East China Normal University

[2]The Key Laboratory of Developmental Genes and Human Disease, School of Life Science and Technology, Southeast University

[3]College of Life Sciences, Nanjing University

[4]School of Data Science, The Chinese University of Hong Kong-Shenzhen

[5]Department of Epidemiology and Biostatistics, School of Public Health, Tongji Medical College, Huazhong University of Science and Technology

1 SpatialAnno method overview

1.1 Probabilistic models for marker and non-marker gene expression

We herein present an overview of SpatialAnno, with its inference details. SpatialAnno requires both spatial transcriptomics data and a list of gene names for known cell/domain-type markers. The marker-gene list can be obtained from either the available publications, databases, or DEGs in scRNA-seq data (see Methods). In the SpatialAnno model, we denote X as the spot-by-gene expression matrix on n spatial locations. These locations have known spatial coordinates and unknown labels y_i, $i = 1, \ldots, n$. We can separate genes into a group of m marker genes and a group of p non-marker genes, denoted as $\boldsymbol{x}_{1i} = (x_{i1}, \ldots, x_{im})^\top$ and $\boldsymbol{x}_{2i} = (x_{i,m+1}, \ldots, x_{i,m+p})^\top$, respectively. Suppose prior knowledge of marker genes for K cell/domain types is encoded as an indicator matrix ρ of dimension $m \times K$, with $\rho_{jk} = 1$ if gene j is a maker for cell/domain type k and 0 otherwise. Following[2,5,16], we assume that the expression measurements have

*Corresponding author, xjshi@fem.ecnu.edu.cn, liujinlab@cuhk.edu.cn.

† The first two authors have contributed equally to this work.

already been normalized through variance stabilizing transformation and further centered for each gene to have zero mean (see Methods).

SpatialAnno models the centered normalized expression vector, $\boldsymbol{x}_{1i}$, for marker genes in cell i, and latent label, y_i, as

$$\begin{aligned} \boldsymbol{x}_{1i} \mid y_i = k &\sim \mathcal{N}(\boldsymbol{\mu}_k, \Sigma), \\ \mu_{jk} &= \alpha_j + \rho_{jk}\beta_{jk}, \end{aligned} \tag{1}$$

with the constraint that $\beta_{jk} \geq 0$. Here, α_j is the base expression level for gene j in the marker group. The intuition is that if gene j is a marker for cell/domain type k, then we expect the expression of j to be higher in these cell/domain types[13] with an increased magnitude β_{jk}. Note that there is no restriction stating maker genes cannot be expressed in other cell/domain types. We assume the covariance $\Sigma = \mathrm{diag}(\sigma_1^2, \ldots, \sigma_m^2)$. This simplification significantly reduces the computational cost.

For the high-dimensional non-marker genes, SpatialAnno models their centered normalized expression vector, $\boldsymbol{x}_{2i}$, and latent label, y_i, as

$$\begin{aligned} \boldsymbol{x}_{2i} \mid \boldsymbol{z}_i &= L\boldsymbol{z}_i + \boldsymbol{e}_i, \\ \boldsymbol{z}_i \mid y_i = k &\sim \mathcal{N}(\boldsymbol{m}_k, V), \end{aligned} \tag{2}$$

where factor $\boldsymbol{z}_i \in R^q$ represents a q-dimensional embedding of $\boldsymbol{x}_{2i}$; L is a $p \times q$ factor loading matrix; $\boldsymbol{m}_k \in R^q$ is the mean vector for the kth cell/domain type, and V is the covariance matrix that is shared across cell/domain types; and $\boldsymbol{e}_i$ is the residual error and follows an independent normal distribution with mean zero and variance Λ, which is a diagonal matrix, or $\boldsymbol{e}_i \sim \mathcal{N}(0, \Lambda)$.

1.2 Potts model for cell/domain labels

In the analysis of SRT datasets, the neighboring locations on the same tissue section often have similar cell/domain types. Thus, spots in neighboring locations contain immense amounts of information for annotating locations of interest. To promote neighborhood similarity in cell/domain types, we follow previous computation[8,12] and assume that cell/domain type $y_i \in \{1, \ldots, K\}$ follows a Potts model characterized by an interaction parameter ξ and a neighborhood graph $\mathcal{S}$,

$$p(\boldsymbol{y} \mid \mathcal{S}, \xi) = \frac{1}{C(\xi)} \exp\left\{ -\xi \sum_{i \sim i'} [1 - \mathrm{I}(y_i = y_{i'})] \right\}, \tag{3}$$

where $i \sim i'$ denotes all neighboring pairs in the neighborhood graph $\mathcal{S}$; $\mathrm{I}(y_i = y_{i'})$ is an indicator function that equals 1 if both the ith and i'th locations belong to the same cell/domain type and equals 0 otherwise; ξ is an unknown interaction parameter that determines the extent of cell/domain type similarity among neighboring locations; and $C(\xi)$ is the normalizing constant, also known as the partition function that ensures the above probability mass function has a summation of one across all possible configurations of $\boldsymbol{y}$.

The hierarchical probabilistic framework of SpatialAnno enables us to develop an efficient optimization algorithm through restricted expectation-maximization (EM)[6] to estimate the

probability of each location of a given cell/domain type. Briefly, our algorithm treats all parameters $\boldsymbol{\theta} = (\{\alpha_j\}, \{\beta_{jk}\}, \{\sigma_j^2\}, \xi, \boldsymbol{m}, V, L, \Lambda)$ as unknown and estimates these parameters based on the data at hand to ensure optimal annotation performance. Algorithm details are provided later.

SpatialAnno has several advantages that facilitate highly accurate assignments and various downstream analyses of spatial transcriptomics. First, by modelling the spatial correlation as labels, SpatialAnno borrows the cell-type information across spatial locations for spatially informed cell/domain type annotation. Second, SpatialAnno models the high-dimensional expression values of non-marker genes with the factor model, which can efficiently utilize the expression of non-marker genes to help verify and adjust label assignments. Third, modelling the high-dimensional expression values of non-marker genes allows SpatialAnno to infer cell-type-relevant embeddings, facilitating effective spatial transcriptomics visualization and spatial trajectory inference.

1.3 Spatial annotation and cell/domain relevant embeddings

To leverage the spatial location information, we construct a neighborhood graph $\mathcal{S}$ among locations by identifying the nearest neighbors for each spot. Specifically, the neighborhood N_i for a spot i is defined by applying a proximity threshold. Let $\boldsymbol{y}_{N_i}$ denote the configurations of the neighbors of spot i. The probability that spot i is associated with cell/domain type k given $\boldsymbol{x}_{i1}$, $\boldsymbol{x}_{i2}$ and its neighbor configuration $\boldsymbol{y}_{N_i}$ is specified by the following equation (see Section 3 for more detials) :

$$\gamma_{ik} = C^{-1} p(\boldsymbol{x}_{i1} \mid y_i = k) p(\boldsymbol{x}_{i2} \mid y_i = k) p(y_i = k \mid \boldsymbol{y}_{N_i}), \tag{4}$$

where C is a normalization constant. In the right-hand side, $p(\boldsymbol{x}_{i1} \mid y_i = k)$ and $p(\boldsymbol{x}_{i2} \mid y_i = k)$ model the effect of the expression levels of marker and non-marker genes, respectively, whereas $p(y_i = k \mid \boldsymbol{y}_{N_i})$ accounts for the effect of the neighbor configuration. The last term is determined by Eq. (3).

A key feature of SpatialAnno is its ability to extract cell/domain relevant embeddings for each spot. By modelling the expression levels of non-marker genes with factor models, SpatialAnno can extract cell/domain-type aware embeddings that can facilitate downstream analyses. Based on Eq. (2) and Bayes' theorem, the conditional distribution of latent factors $\boldsymbol{z}_i$ given $(\boldsymbol{x}_{i1}, \boldsymbol{x}_{i2}, y_i = k)$ follows a multivariate normal distribution $\mathcal{N}(\boldsymbol{w}_{ik}, M)$ with mean $\boldsymbol{w}_{ik}$ and variance M (see Section 3 for more details). The low-dimensional embeddings for spot i are estimated by the posterior expectation of its latent factors $\boldsymbol{z}_i$:

$$\mathrm{E}(\boldsymbol{z}_i \mid \boldsymbol{x}_{i1}, \boldsymbol{x}_{i2}, \boldsymbol{y}_{N_i}) = \sum_k \gamma_{ik} \boldsymbol{w}_{ik}, \tag{5}$$

which are weighted averages that take into account the relative importance of each type. In this way, the embeddings are encouraged to be label-relevant.

2 Details on simulations

The spatial locations of 3639 spots were taken from DLPFC section 151673. Cell/domain types were assigned with manually generated annotations from the original studies[9]. We simulated

gene expression data for each spot using the *splatter* package (version 1.20.0). The parameter for the proportion of DEGs (*de.prob*) in each layer was set to 0.5. The DE strength was determined by both the mean parameter *de.facloc* and scale parameter *de.facScale*, the former ranges from 0.1 to 0.8, and the latter was set to within [0.1,1], corresponding to the log fold change in expression from one-fold to two-fold across different types. All the other parameters were set based on their estimates in the seven layers from DLPFC section 151673.

3 Details on data analysis

3.1 Normalization of SRT data

For all datasets, we normalized the raw expression count matrix using the variance stabilizing transformation function, `SCTranscorm`, provided in *Seurat* (version 4.1.1). We performed gene filtering using *SPARK* (version 1.1.1)[17] for data with transcriptome-wide measurements. The most spatially variable genes (see Data resource) were selected as input for the annotation methods SpatialAnno, scSorter, SCINA, and Garnett. CellAssign took the raw count matrix of these genes as input.

3.2 Selection of marker genes

We obtained a marker gene list primarily following the protocols of CellAssign. We (1) performed differential expression analysis of a reference scRNA-seq/SRT data using the `FindAllMarkers` function in the R package *Seurat* (version 4.1.1) and selected the top 30 DEGs ordered by the log2(fold-change) with upregulation, (2) removed those with an insignificant adjusted p-value and those detection percentages across different cell/domain types were similar (differences between pct.1 and pct.2 values from the `FindAllMarkers` function are lower than 0.3), and (3) filtered out genes that were of low expression in the spatial transcriptomic data. We finally selected the top-ranked genes with the smallest p-values.

3.3 Clustering analysis

To examine the information captured by SpaitalAnno embeddings of non-marker genes, we performed clustering analysis using three different sets of embeddings as input in both the simulated and DLPFC data. The three embedding sets include the top 15 PCs in marker genes by PCA, 15-dimensional embeddings in non-marker genes by SpatialAnno, and combined. We then performed clustering analysis using the Louvain community detection algorithm.

3.4 Spatial trajectory inference

To construct a spatial map of the DLPFC Visium data, we employed the PAGA algorithm[11] implemented in the Python package *SCANPY* (version 1.9.1) to preserve the global topology in the embeddings of non-marker genes. The cluster labels for PCA embeddings and DR-SC embeddings were estimated using the spatial clustering methods implemented in the R packages *BayesSpace* (version 1.5.1)[15] and *DR-SC* (version 2.9.0)[8], respectively.

To estimate the developmental trajectories among the various locations in the brain regions, we applied Slingshot[10] to the low-dimensional embeddings. As SpatialAnno only extracts embeddings of non-marker genes, we combined them with the embeddings of marker genes by PCA. The cluster labels used in Slingshot were obtained from the spatial clustering method DR-SC[8]. To detect DEGs along the estimated pseudotime, we used the function `testPseudotime` in the R package *TSCAN* (version 1.37.0)[7].

4 Details on evaluations in both simulations and real data sets

Cohen's kappa value (Kappa) is generally thought to be a more robust measure than classification accuracy (ACC), as it takes into account the possibility of the agreement occurring by chance. The definition of Kappa is

$$\text{Kappa} = \frac{\text{ACC} - p_e}{1 - p_e},$$

where p_e is the hypothetical probability of chance agreement. For K cell types, N cells to annotate, and n_k is the number of cells belonging to cell type k in the ground truth:

$$p_e = \frac{1}{N^2} \sum a_k n_k,$$

where a_k is the number of the k-th cell type assigned by some annotation method. A Kappa of 1 implies the annotation results are in complete agreement with the ground truth, whereas 0 implies that the annotation is no better than a random guess.

mF1 is the average of F1 scores for different cell types. The cell-type-level F1 score considers each cell as an individual classification task with a true cell-type assignment. We calculated the F1 score as follows:

$$\text{F1 score} = 2 \times \frac{\text{precision} \times \text{recall}}{\text{precision} + \text{recall}},$$

where precision is the number of true positive samples divided by the number of all samples identified as this cell type, and recall is the number of true positive samples divided by the number of samples in this cell type.

5 Statiscal inference of SpatialAnno

Statistical inference for SpatialAnno is done using a restricted expectation-maximization algorithm[6] with an iterative conditional mode[3]. The latent variables are $(\boldsymbol{y}, \boldsymbol{z})$, and the model parameters to be maximized are $\boldsymbol{m} = \{\boldsymbol{m}_k\}$, V, L, Λ, $\boldsymbol{\alpha} = \{\alpha_j\}$, $\boldsymbol{\beta} = \{\beta_{jk}\}$, $\boldsymbol{\sigma} = \{\sigma_j\}$, and ξ. To facilitate description, we denote $\boldsymbol{\theta}_1 = \{\boldsymbol{\alpha}, \boldsymbol{\beta}, \boldsymbol{\sigma}\}$ as the parameters in equation (1) from the main text and $\boldsymbol{\theta}_2 = \{\boldsymbol{m}, V, L, \Lambda\}$ as the parameters in equation (2) from the main text.

The complete data likelihood $p(\boldsymbol{x}, \boldsymbol{y}, \boldsymbol{z}) = p(\boldsymbol{x}, \boldsymbol{z}, \mid \boldsymbol{y})p(\boldsymbol{y})$ is difficult to deal with because of the complicated form of $p(\boldsymbol{y})$. To overcome the bottleneck, one of the most used approaches is pseudo likelihood[1], which approximates the joint distribution of $\boldsymbol{y}$ as the product of the

full-conditional distribution for each y_i. Therefore, the pseudo log-likelihood function of the complete-data is

$$\begin{aligned}\log \tilde{p}(\boldsymbol{x}, \boldsymbol{y}, \boldsymbol{z}) &= \log p(\boldsymbol{x}, \boldsymbol{z} \mid \boldsymbol{y}) + \log \tilde{p}(\boldsymbol{y}) \\ &= \sum_i \log p(\boldsymbol{x}_{i1}, \boldsymbol{x}_{i2}, \boldsymbol{z}_i \mid y_i) + \sum_i \log p(y_i \mid y_{N_i}) \\ &= \sum_i \log p(\boldsymbol{x}_{i1} \mid y_i) + \sum_i \log p(\boldsymbol{x}_{i2}, \boldsymbol{z}_i \mid y_i) + \sum_i \log p(y_i \mid y_{N_i}) \\ &= \sum_i \log p(\boldsymbol{x}_{i1}, \boldsymbol{x}_{i2}, \boldsymbol{z}_i, y_i \mid \boldsymbol{y}_{N_i}).\end{aligned} \tag{6}$$

Starting at some initial values for $\boldsymbol{y}$ and parameters $\boldsymbol{\theta} = \{\boldsymbol{\theta}_1, \boldsymbol{\theta}_2, \xi\}$, the algorithm iterates through an iterative conditional mode (ICM) step, expectation step (E-step), and maximization step (M-step) to maximize the pseudo log-likelihood. The initial values could be provided by a non-spatial annotation method. For transcriptome-wide SRT data (such as ST and Visium), we relied on SCINA[14] because of its computational efficiency. For SRT data with limited multiplexing capacity (such as from seqFISH), scSorter[4] can also be used. Alternative initialization can also be supplied by users. We describe the detailed optimization algorithm for each step in the following subsections.

5.1 ICM-step: updating $\boldsymbol{y}$

Based on the Potts model, we know

$$\log p(y_i \mid \boldsymbol{y}_{N_i}) = -\log C_\xi(\boldsymbol{y}_{N_i}) - \xi \sum_{i' \in N_i} \left[1 - \mathrm{I}(y_i = y_{i'})\right],$$

where $C_\xi(\boldsymbol{y}_{N_i})$ is a normalization constant with respect to ξ and $\boldsymbol{y}_{N_i}$.

Based on the properties of the normal distribution, we know $\boldsymbol{x}_{i2} \mid y_i = k$ is a normal, and its mean and variance can be derived from the law of total expectation:

$$\begin{aligned}\mathrm{E}(\boldsymbol{x}_{i2} \mid y_i = k) &= \mathrm{E}\left[\mathrm{E}(\boldsymbol{x}_{i2} \mid y_i = k, \boldsymbol{z}_i) \mid y_i = k\right] \\ &= \mathrm{E}\left[\mathrm{E}(\boldsymbol{x}_{i2} \mid \boldsymbol{z}_i) \mid y_i = k\right] \\ &= \mathrm{E}(L\boldsymbol{z}_i \mid y_i = k) \\ &= L\boldsymbol{\mu}_k, \\ \mathrm{Var}(\boldsymbol{x}_{i2} \mid y_i = k) &= \mathrm{E}\left[\mathrm{Var}(\boldsymbol{x}_i \mid \boldsymbol{z}_i) \mid y_i = k\right] + \mathrm{Var}\left[\mathrm{E}(\boldsymbol{x}_i \mid \boldsymbol{z}_i) \mid y_i = k\right] \\ &= \mathrm{E}\left(\Lambda \mid y_i = k\right) + \mathrm{Var}\left(L\boldsymbol{z}_i \mid y_i = k\right) \\ &= \Lambda + LVL^\top.\end{aligned} \tag{7}$$

We have

$$\log p(\boldsymbol{x}_{i2} \mid y_i = k) = -\frac{p}{2}\log 2\pi + \frac{1}{2}\log|S| - \frac{1}{2}(\boldsymbol{x}_{i2} - L\boldsymbol{\mu}_k)^\top S(\boldsymbol{x}_{i2} - L\boldsymbol{\mu}_k),$$

where $S = (\Lambda + LVL^\top)^{-1}$. To calculate S efficiently, we can use the Woodbury formula:

$$S = \Lambda^{-1} - \Lambda^{-1}LML^\top\Lambda^{-1}.$$

where $M = (V^{-1} + L^\top \Lambda^{-1} L)^{-1}$.

In the ICM step, the estimate of $\boldsymbol{y}$ is obtained by maximizing its posterior with respect to y_i coordinately:

$$p(\boldsymbol{y} \mid \boldsymbol{x}) = p(y_i, \boldsymbol{y}_{-i} \mid \boldsymbol{x}) = p(y_i \mid \boldsymbol{x}, \boldsymbol{y}_{-i}) p(\boldsymbol{y}_{-i} \mid \boldsymbol{x}),$$

where $i = 1, \ldots, n$, until converge. As the second term in the last equation does not depend on y_i, and

$$p(y_i \mid \boldsymbol{x}, \boldsymbol{y}_{-i}) \propto p(\boldsymbol{x}_i \mid y_i) p(y_i \mid \boldsymbol{y}_{N_i} = \hat{\boldsymbol{y}}_{N_i}),$$

we have

$$\begin{aligned}
\hat{y}_i^u =& \arg\max_{y_i} \left[\log p(\boldsymbol{x}_i \mid y_i) + \log p(y_i \mid \boldsymbol{y}_{N_i} = \hat{\boldsymbol{y}}_{N_i})\right] \\
=& \arg\max_{y_i} \left[\log p(\boldsymbol{x}_{1i} \mid y_i) + \log p(\boldsymbol{x}_{2i} \mid y_i) + \log p(y_i \mid \boldsymbol{y}_{N_i} = \hat{\boldsymbol{y}}_{N_i})\right] \\
=& \arg\min_k \left\{ \sum_{j=1}^m \left[\frac{1}{2} \log 2\pi + \frac{1}{2} \log \sigma_j^2 + \frac{1}{2\sigma_j^2} (x_{ij} - \alpha_j - \rho_{jk} \beta_{jk})^2 \right] \right. \\
& + \frac{p}{2} \log 2\pi - \frac{1}{2} \log |S| + \frac{1}{2} (\boldsymbol{x}_{i2} - L\boldsymbol{\mu}_k)^\top S (\boldsymbol{x}_{i2} - L\boldsymbol{\mu}_k) \\
& \left. + \log C_\xi(\hat{\boldsymbol{y}}_{N_i}) + \xi \sum_{i' \in N_i} [1 - \mathrm{I}(\hat{y}_{i'} = k)] \right\}.
\end{aligned} \tag{8}$$

5.2 E-step: updating responsibility

Now, we define the responsibility that component k takes for explaining the observation $\boldsymbol{x}_i$ as

$$\begin{aligned}
\gamma_{ik} =& p(y_i = k \mid \boldsymbol{x}_i, \boldsymbol{y}_{N_i} = \hat{\boldsymbol{y}}_{N_i}) \\
=& \frac{p(\boldsymbol{x}_i \mid y_i = k) p(y_i = k \mid \boldsymbol{y}_{N_i} = \hat{\boldsymbol{y}}_{N_i})}{\sum_{k'} p(\boldsymbol{x}_i \mid y_i = k') p(y_i = k') \mid \boldsymbol{y}_{N_i} = \hat{\boldsymbol{y}}_{N_i})} \\
=& \frac{p(\boldsymbol{x}_{i1} \mid y_i = k) p(\boldsymbol{x}_{i2} \mid y_i = k) p(y_i = k \mid \boldsymbol{y}_{N_i} = \hat{\boldsymbol{y}}_{N_i})}{\sum_{k'} p(\boldsymbol{x}_{i1} \mid y_i = k') p(\boldsymbol{x}_{i2} \mid y_i = k') p(y_i = k') \mid \boldsymbol{y}_{N_i} = \hat{\boldsymbol{y}}_{N_i})}.
\end{aligned} \tag{9}$$

It is easy to show that the optimal posterior distribution of $(y_i = k, \boldsymbol{z}_i)$ is

$$\begin{aligned}
p(y_i = k, \boldsymbol{z}_i \mid \boldsymbol{x}_i, \boldsymbol{y}_{N_i} = \hat{\boldsymbol{y}}_{N_i}) &= p(y_i = k \mid \boldsymbol{x}_i, \boldsymbol{y}_{N_i} = \hat{\boldsymbol{y}}_{N_i}) p(\boldsymbol{z}_i \mid \boldsymbol{x}_i, y_i = k) \\
&= \gamma_{ik} \mathcal{N}(\boldsymbol{z}_i \mid \boldsymbol{w}_{ik}, M),
\end{aligned} \tag{10}$$

where

$$\begin{aligned}
\boldsymbol{w}_{ik} &= M \left[V^{-1} \boldsymbol{m}_k + L^\top \Lambda^{-1} \boldsymbol{x}_{i2} \right], \\
M &= \left[V^{-1} + L^\top \Lambda^{-1} L \right]^{-1}.
\end{aligned} \tag{11}$$

Note that the conditional expectation of $\boldsymbol{z}_i$ given $(\boldsymbol{x}_i, \boldsymbol{y}_{N_i} = \hat{\boldsymbol{y}}_{N_i})$ provides a low-dimensional embedding for spot i:

$$\mathrm{E}(\boldsymbol{z}_i \mid \boldsymbol{x}_i, \boldsymbol{y}_{N_i} = \hat{\boldsymbol{y}}_{N_i}) = \sum_k \gamma_{ik} \boldsymbol{w}_{ik}.$$

Obviously, the embedding is label-relevant due to γ_{ik}.

5.3 Restricted M-step

Taking expectation of (6) w.r.t. the posterior distribution of $(y_i, \boldsymbol{z}_i)$ in (10), the Q function is

$$
\begin{aligned}
Q(\boldsymbol{\theta}) &= \sum_i \mathrm{E}_{q_i} \log\left[p(\boldsymbol{x}_i, \boldsymbol{z}_i, y_i \mid \boldsymbol{y}_{N_i} = \hat{\boldsymbol{y}}_{N_i})\right] \\
&= \sum_i \sum_k \int \log\left[p(\boldsymbol{x}_i, \boldsymbol{z}_i, y_i = k \mid \boldsymbol{y}_{N_i} = \hat{\boldsymbol{y}}_{N_i})\right] p(y_i = k, \boldsymbol{z}_i \mid \boldsymbol{x}_i, \boldsymbol{y}_{N_i} = \hat{\boldsymbol{y}}_{N_i}) d\boldsymbol{z}_i \\
&= \sum_i \sum_k \int \gamma_{ik} \left[\log p(\boldsymbol{x}_i, \boldsymbol{z}_i \mid y_i = k) + \log p(y_i = k \mid \boldsymbol{y}_{N_i} = \hat{\boldsymbol{y}}_{N_i})\right] \mathcal{N}(\boldsymbol{w}_{ik}, M) d\boldsymbol{z}_i \\
&= \sum_i \sum_k \int \gamma_{ik} \left[\log p(\boldsymbol{x}_i \mid \boldsymbol{z}_i) + \log p(\boldsymbol{z}_i \mid y_i = k) + \log p(y_i = k \mid \boldsymbol{y}_{N_i} = \hat{\boldsymbol{y}}_{N_i})\right] \mathcal{N}(\boldsymbol{w}_{ik}, M) d\boldsymbol{z}_i \\
&= \sum_i \sum_{j=1}^m \sum_k \gamma_{ik} \left[-\frac{1}{2}\log 2\pi - \frac{1}{2}\log \sigma_j^2 - \frac{1}{2\sigma_j^2}(x_{ij} - \alpha_j - \rho_{jk}\beta_{jk})^2\right] \\
&\quad + \sum_i \sum_k \gamma_{ik} \left[-\frac{p}{2}\log 2\pi - \frac{1}{2}\log|\Lambda| - \frac{1}{2}(\boldsymbol{x}_{2i} - L\boldsymbol{w}_{ik})^\top \Lambda^{-1}(\boldsymbol{x}_{2i} - L\boldsymbol{w}_{ik}) - \frac{1}{2}\mathrm{Tr}(\Lambda^{-1}LML^\top)\right] \\
&\quad + \sum_i \sum_k \gamma_{ik} \left[-\frac{q}{2}\log 2\pi - \frac{1}{2}\log|V| - \frac{1}{2}(\boldsymbol{w}_{ik} - \boldsymbol{m}_k)^\top V^{-1}(\boldsymbol{w}_{ik} - \boldsymbol{m}_k) - \frac{1}{2}\mathrm{Tr}(V^{-1}M)\right] \\
&\quad - \sum_i \log C_i(\xi, \hat{\boldsymbol{y}}_{N_i}) - \xi \sum_i \sum_k \gamma_{ik} \sum_{i' \in N_i} \left[1 - \mathrm{I}(y_{i'} = k)\right],
\end{aligned} \tag{12}
$$

5.3.1 Updating $\boldsymbol{\theta}_1$

The parameter $\beta_{jk} \geq 0$ corresponds to the average fold-change in the expression of gene j overexpressed in type k. In real data applications, where well-understood marker genes exist, a *priori* information about their expression levels in corresponding cell types may be available. To incorporate such information, other lower boundaries can be imposed.

As there is a linear inequality constraining $\beta_{jk} \geq 0$, we performed constrained optimization via the following Lagarangian

$$Q(\boldsymbol{\theta}) + \sum_j \sum_k \eta_{jk}\rho_{jk}\beta_{jk}.$$

The Karush-Kuhn-Tucker (KKT) conditions were

$$
\begin{aligned}
\rho_{jk}\beta_{jk} &\geq 0, \\
\eta_{jk} &\geq 0, \\
\eta_{jk}\rho_{jk}\beta_{jk} &= 0, \\
\frac{\partial Q}{\partial \beta_{jk}} &= \sum_i \frac{\gamma_{ik}}{\sigma_j^2}(x_{ij} - \alpha_j - \rho_{jk}\beta_{jk})\rho_{jk} + \eta_{jk}\rho_{jk} = 0 \text{ for } \rho_{jk} = 1, \\
\frac{\partial Q}{\partial \alpha_j} &= \sum_i \sum_k \frac{\gamma_{ik}}{\sigma_j^2}(x_{ij} - \alpha_j - \rho_{jk}\beta_{jk}) = 0, \\
\frac{\partial Q}{\partial \sigma_j^2} &= \sum_i \sum_k \gamma_{ik}\left[-\frac{1}{2\sigma_j^2} + \frac{1}{2\sigma_j^4}(x_{ij} - \alpha_j - \rho_{jk}\beta_{jk})^2\right].
\end{aligned} \tag{13}
$$

Examining the KKT conditions will show the final solution to be

$$\begin{aligned}\alpha_j =& \frac{1}{n}\sum_i\sum_k \gamma_{ik}(x_{ij}-\rho_{jk}\beta_{jk}),\\ \beta_{jk} =& \max\left\{\frac{\sum_i \gamma_{ik}(x_{ij}-\alpha_j)}{\sum_i \gamma_{ik}}, 0\right\} \text{ for } \rho_{jk}=1,\\ \sigma_j^2 =& \frac{1}{n}\sum_i\sum_k \gamma_{ik}(x_{ij}-\alpha_j-\rho_{jk}\beta_{jk})^2.\end{aligned} \tag{14}$$

5.3.2 Updating $\boldsymbol{\theta}_2$

By setting the first partial derivative of $Q(\theta)$ w.r.t $\boldsymbol{\theta}_2$ to zero, we obtained the updates for all parameters as follows:

$$\begin{aligned}\boldsymbol{m}_k =& \frac{\sum_i \gamma_{ik}\boldsymbol{w}_{ik}}{\sum_i \gamma_{ik}},\\ V =& \frac{\sum_i\sum_k \gamma_{ik}(\boldsymbol{w}_{ik}-\boldsymbol{m}_k)(\boldsymbol{w}_{ik}-\boldsymbol{m}_k)^\top}{\sum_i\sum_k \gamma_{ik}} + M,\\ L =& \left[\sum_i\sum_k \gamma_{ik}\boldsymbol{x}_i\boldsymbol{w}_{ik}^\top\right]\left[\sum_i\sum_k \gamma_{ik}\boldsymbol{w}_{ik}\boldsymbol{w}_{ik}^\top + nM\right]^{-1},\\ \lambda_j =& \frac{1}{n}\sum_i\sum_k \gamma_{ik}(\boldsymbol{x}_{ij}-L_j^\top\boldsymbol{w}_{ik})^2 + L_j^\top M L_j.\end{aligned} \tag{15}$$

5.3.3 Updating ξ

Taking the first derivative of $Q(\boldsymbol{\theta})$ w.r.t as the parameter ξ in the Potts model is difficult because of the normalization constant $C(\xi)$, which requires evaluating the probability mass function of the Potts model over all possible configurations of $\boldsymbol{y}$, and it is thus known to be NP hard. We optimized it numerically via a grid search strategy:

$$\xi = \arg\max_{l\in 1,\ldots,R} Q(\boldsymbol{\theta}_1, \boldsymbol{\theta}_2, \xi_l),$$

where the sequence $\xi_1, \ldots, \xi_R$ is a vector of evenly spaced points in the interval $[0, \xi_{\max}]$. We set the upper bound $\xi_{\max}$ in the uniform distribution to be a large number (set to be 2.5 here) representing the other extreme cases in which spatial location information is highly informative and in which the resulting spatial domain boundaries are extremely smooth.

References

[1] Julian Besag. On the statistical analysis of dirty pictures. *Journal of the Royal Statistical Society: Series B (Methodological)*, 48(3):259–279, 1986.

[2] Andrew Butler, Paul Hoffman, Peter Smibert, Efthymia Papalexi, and Rahul Satija. Integrating single-cell transcriptomic data across different conditions, technologies, and species. *Nature biotechnology*, 36(5):411–420, 2018.

[3] Meritxell Bach Cuadra, Leila Cammoun, Torsten Butz, Olivier Cuisenaire, and J-P Thiran. Comparison and validation of tissue modelization and statistical classification methods in t1-weighted mr brain images. *IEEE transactions on medical imaging*, 24(12):1548–1565, 2005.

[4] Hongyu Guo and Jun Li. scsorter: assigning cells to known cell types according to marker genes. *Genome biology*, 22(1):1–18, 2021.

[5] Christoph Hafemeister and Rahul Satija. Normalization and variance stabilization of single-cell rna-seq data using regularized negative binomial regression. *Genome biology*, 20(1):1–15, 2019.

[6] Mortaza Jamshidian. On algorithms for restricted maximum likelihood estimation. *Computational statistics & data analysis*, 45(2):137–157, 2004.

[7] Zhicheng Ji and Hongkai Ji. Tscan: Pseudo-time reconstruction and evaluation in single-cell rna-seq analysis. *Nucleic acids research*, 44(13):e117–e117, 2016.

[8] Wei Liu, Xu Liao, Yi Yang, Huazhen Lin, Joe Yeong, Xiang Zhou, Xingjie Shi, and Jin Liu. Joint dimension reduction and clustering analysis of single-cell rna-seq and spatial transcriptomics data. *Nucleic acids research*, 50(12):e72–e72, 2022.

[9] Kristen R Maynard, Leonardo Collado-Torres, Lukas M Weber, Cedric Uytingco, Brianna K Barry, Stephen R Williams, Joseph L Catallini, Matthew N Tran, Zachary Besich, Madhavi Tippani, et al. Transcriptome-scale spatial gene expression in the human dorsolateral prefrontal cortex. *Nature neuroscience*, 24(3):425–436, 2021.

[10] Kelly Street, Davide Risso, Russell B Fletcher, Diya Das, John Ngai, Nir Yosef, Elizabeth Purdom, and Sandrine Dudoit. Slingshot: cell lineage and pseudotime inference for single-cell transcriptomics. *BMC genomics*, 19(1):1–16, 2018.

[11] F Alexander Wolf, Fiona K Hamey, Mireya Plass, Jordi Solana, Joakim S Dahlin, Berthold Göttgens, Nikolaus Rajewsky, Lukas Simon, and Fabian J Theis. Paga: graph abstraction reconciles clustering with trajectory inference through a topology preserving map of single cells. *Genome biology*, 20(1):1–9, 2019.

[12] Yi Yang, Xingjie Shi, Wei Liu, Qiuzhong Zhou, Mai Chan Lau, Jeffrey Chun Tatt Lim, Lei Sun, Cedric Chuan Young Ng, Joe Yeong, and Jin Liu. Sc-meb: spatial clustering with hidden markov random field using empirical bayes. *Briefings in bioinformatics*, 23(1):bbab466, 2022.

[13] Allen W Zhang, Ciara O'Flanagan, Elizabeth A Chavez, Jamie LP Lim, Nicholas Ceglia, Andrew McPherson, Matt Wiens, Pascale Walters, Tim Chan, Brittany Hewitson, et al. Probabilistic cell-type assignment of single-cell rna-seq for tumor microenvironment profiling. *Nature methods*, 16(10):1007–1015, 2019.

[14] Ze Zhang, Danni Luo, Xue Zhong, Jin Huk Choi, Yuanqing Ma, Stacy Wang, Elena Mahrt, Wei Guo, Eric W Stawiski, Zora Modrusan, et al. Scina: a semi-supervised subtyping algorithm of single cells and bulk samples. *Genes*, 10(7):531, 2019.

[15] Edward Zhao, Matthew R Stone, Xing Ren, Jamie Guenthoer, Kimberly S Smythe, Thomas Pulliam, Stephen R Williams, Cedric R Uytingco, Sarah EB Taylor, Paul Nghiem, et al. Spatial transcriptomics at subspot resolution with bayesspace. *Nature Biotechnology*, 39(11):1375–1384, 2021.

[16] Grace XY Zheng, Jessica M Terry, Phillip Belgrader, Paul Ryvkin, Zachary W Bent, Ryan Wilson, Solongo B Ziraldo, Tobias D Wheeler, Geoff P McDermott, Junjie Zhu, et al. Massively parallel digital transcriptional profiling of single cells. *Nature communications*, 8(1):1–12, 2017.

[17] Jiaqiang Zhu, Shiquan Sun, and Xiang Zhou. Spark-x: non-parametric modeling enables scalable and robust detection of spatial expression patterns for large spatial transcriptomic studies. *Genome biology*, 22(1):1–25, 2021.
