# Supplementary material for: Probabilistic cell/domain-type assignment of spatial transcriptomics data with SpatialAnno
Source: Nucleic Acids Res. 2023 Nov 6;51(22):e115. doi: 10.1093/nar/gkad1023 (PMC10711557; doi:10.1093/nar/gkad1023)
Supplement: gkad1023_Supplemental_Files [file gkad1023_supplemental_files.zip › 5 supplementary information.pdf]

Supplementary Information

Probabilistic cell/domain-type assignment of spatial transcriptomics data with SpatialAnno

Xingjie Shi[1,†,*], Yi Yang[2,†], Xiaohui Ma[3], Yong Zhou[1], Zhenxin Guo[4], Chaolong Wang[5], Jin Liu[4,*]

1. KLATASDS-MOE, Academy of Statistics and Interdisciplinary Sciences, School of Statistics, East China Normal University
2. The Key Laboratory of Developmental Genes and Human Disease, School of Life Science and Technology, Southeast University
3. College of Life Sciences, Nanjing University
4. School of Data Science, The Chinese University of Hong Kong, Shenzhen
5. Department of Epidemiology and Biostatistics, School of Public Health, Tongji Medical College, Huazhong University of Science and Technology

Supplementary Figures

Supplementary Figure 1. Additional simulation results.

a Bayes factors to compare the performance (Kappa, mF1 and ACC) of each method against SpatialAnno, providing different number of cell/domain types with marker genes as input. Bayes factors greater than 20 were truncated to 20. **b** As in a, with various degrees of mis-specification in marker genes. **c** Boxplots of Kappa, mF1 and ACC of different methods with various proportions of overlapped marker genes. **d** As in a,with various degrees of overlapped maker genes.

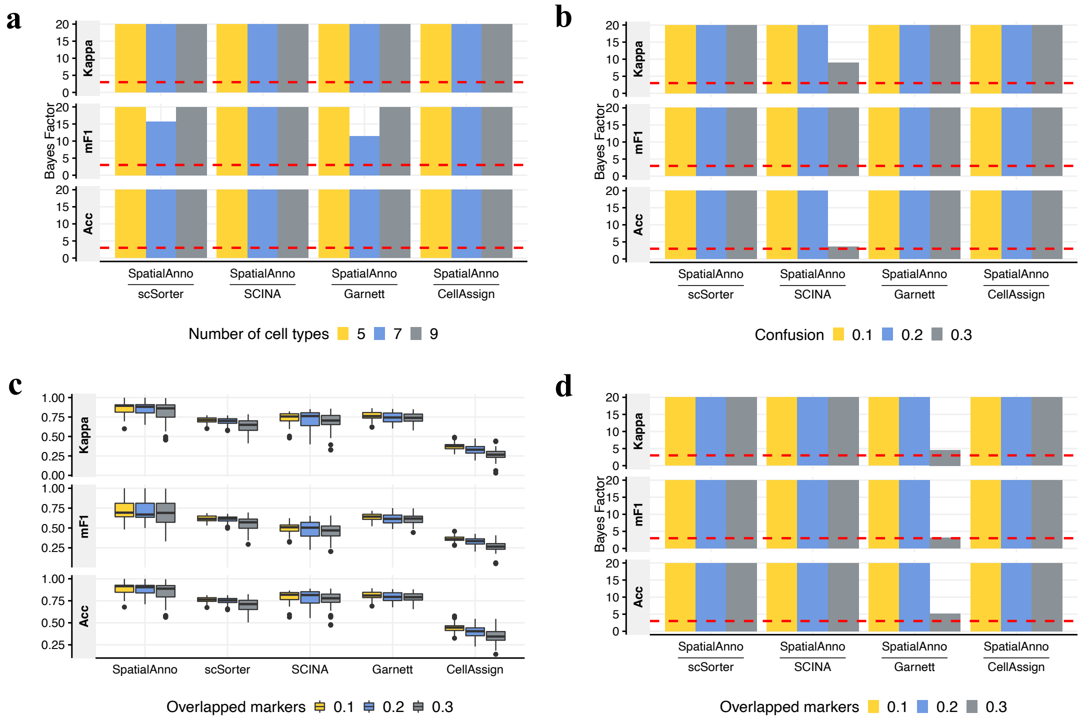

Supplementary Figure 2. Additional simulation results

a Kappa, mF1 and ACC of SpatialAnno, scSorter, and Garnett for simulation data, with different numbers of non-marker genes provided as input. Two-sided Wilcoxon Rank Sum test was used to pair wisely test the difference between metrices with 60 and 2000 non-marker genes, and the *p*-value is shown. **b** Clustering results measured by adjusted rand index (ARI, the higher the better) using low-dimensional embeddings either from marker genes by PCA or non-marker genes by SpatialAnno, or combined for simulation data, providing different number of cell/domain types with marker genes as input. **c** As in **b**, with various degrees of mis-specification in marker genes. **d** Pearson's correlation coefficients between observed expression and the inferred labels, conditioned on embeddings from SpatialAnno, PCA, and DR-SC, providing different number of cell/domain types with marker genes as input. **e** As in **d**, with various degrees of mis-specification in marker genes. **f** Runtime (in seconds) benchmarking of different methods with different number of cell/domain types.

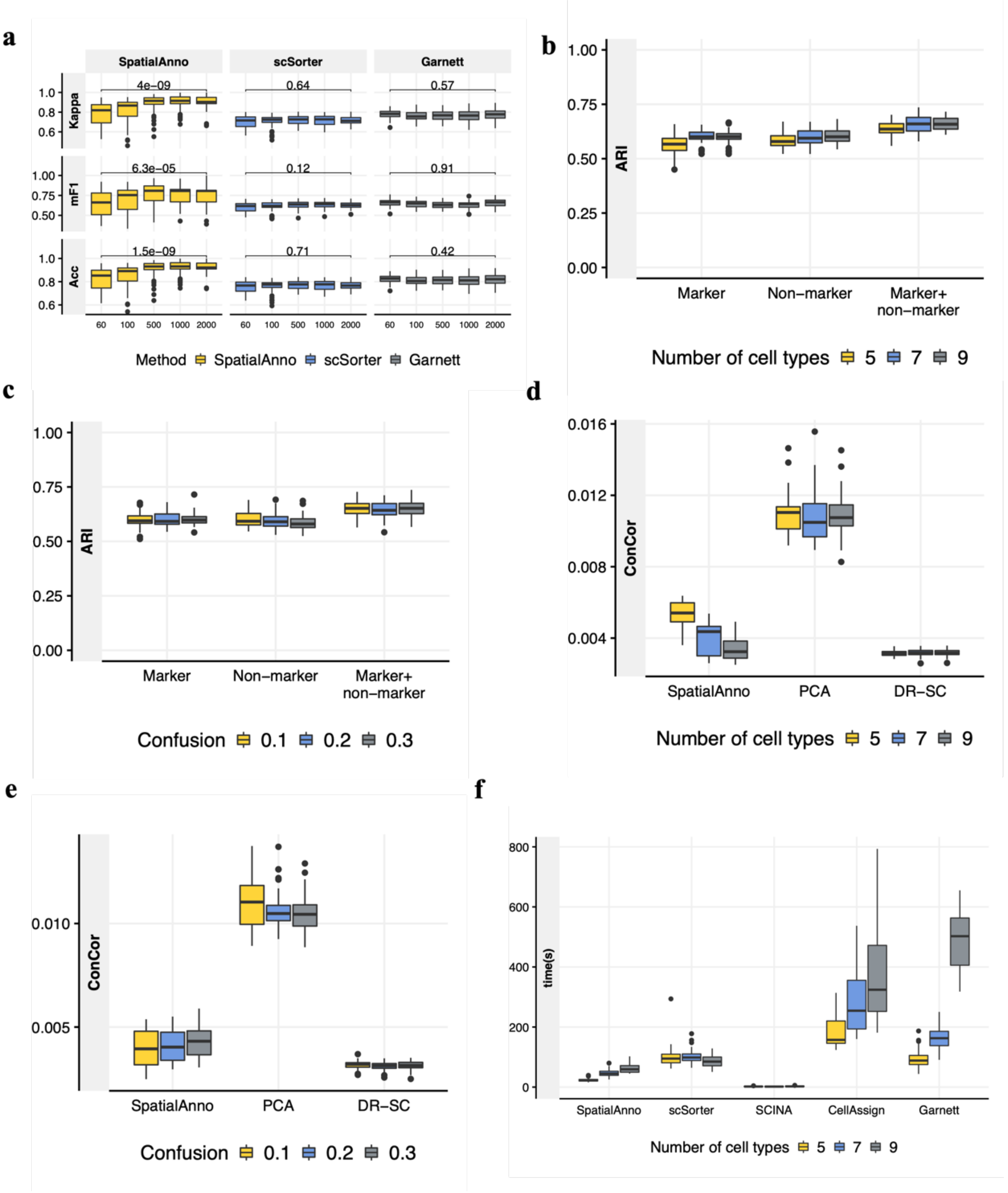

Supplementary Figure 3. Bayes factors to compare the performance of each method against SpatialAnno in the DLPFC 10x Visium dataset. Bayes factors greater than 50 were truncated to 50. A Bayes factor greater than 3 was considered statistically different.

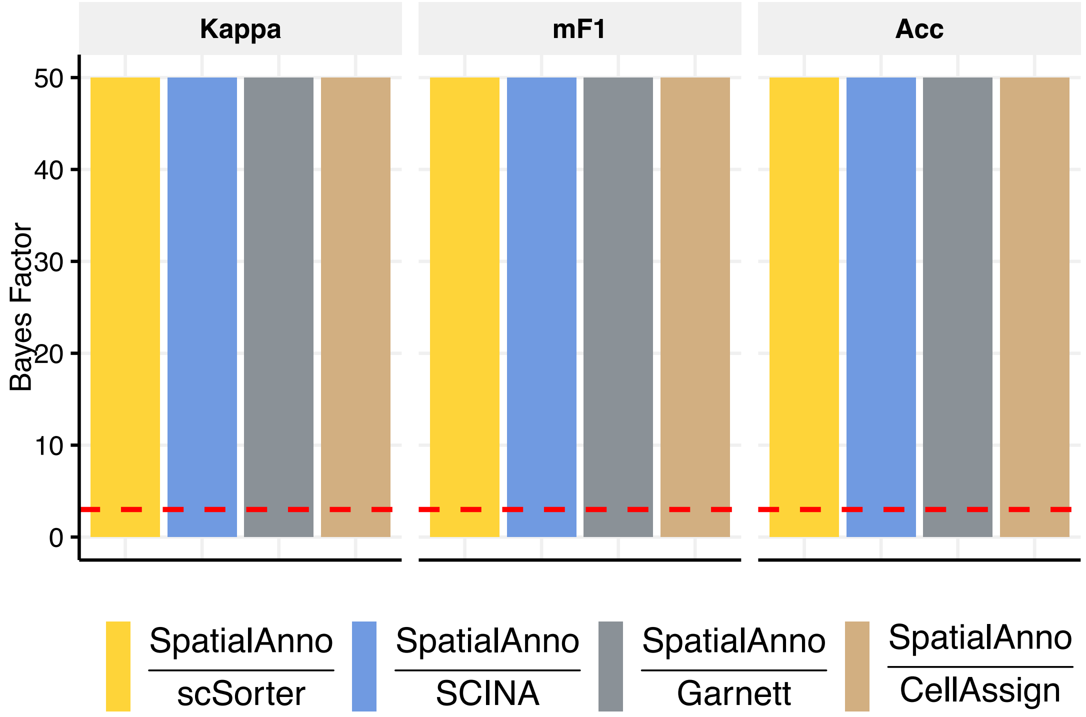

Supplementary Figure 4. Spatial domain annotation in the DLPFC section 151507
a Spatial domain annotations of tissue section 151507 are shown for ground truth, SpatialAnno, scSorter, SCINA, Garnett, and CellAssign. **b** Top, annotation by SpatialAnno for each spot. Bottom, expression levels of corresponding layer-specific marker genes. **c** Top, RGB plots for low-dimensional embedding inferred by SpatialAnno, PCA, and DR-SC. As end-to-end annotation approaches, scSorter, SCINA, Garnett, and CellAssign cannot be utilized to extract low-dimensional embedding. Bottom, PAGA graphs generated by SpatialAnno, PCA, and DR-SC embeddings.

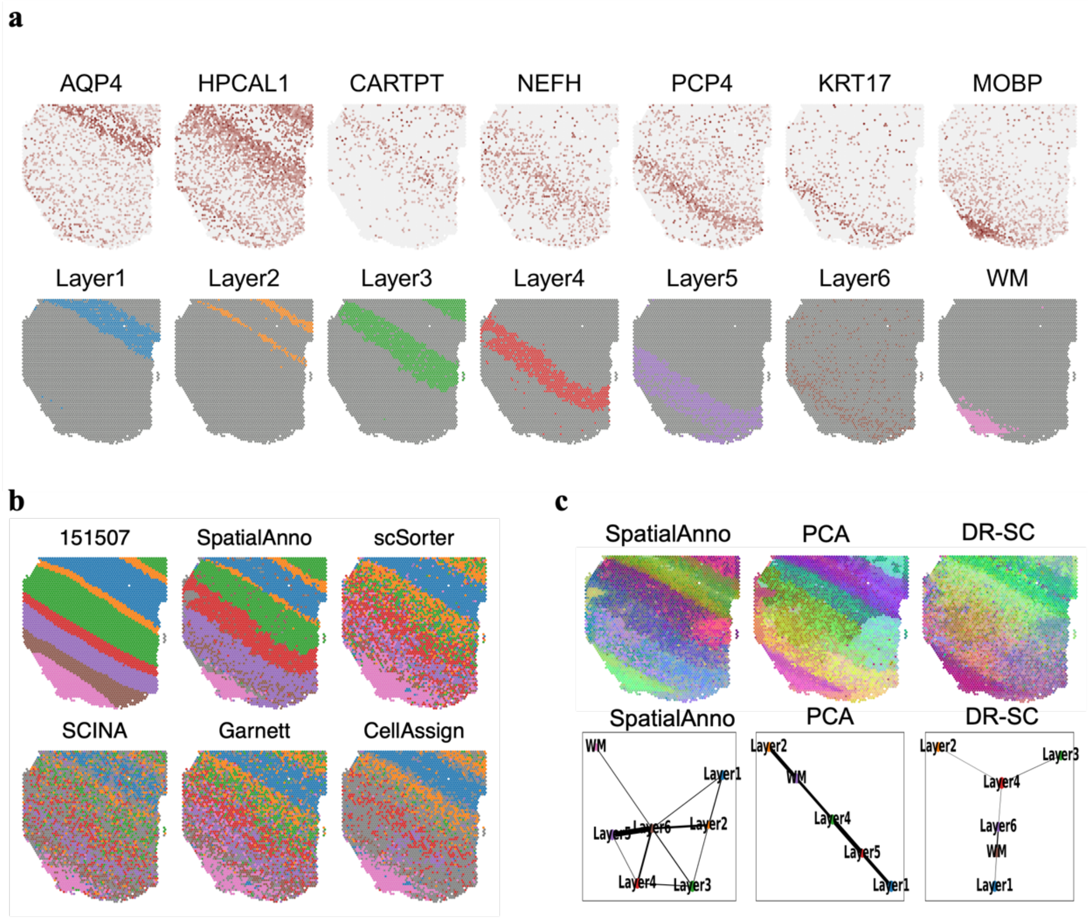

Supplementary Figure 5. Spatial domain annotation of DLPFC section 151508

a Spatial domain annotations of tissue section 151508 are shown for ground truth, SpatialAnno, scSorter, SCINA, Garnett, and CellAssign. **b** Top, annotation by SpatialAnno for each spot. Bottom, expression levels of corresponding layer-specific marker genes. **c** Top, RGB plots for the low dimensional embedding inferred by SpatialAnno, PCA, and DR-SC. As end-to-end annotation approaches, scSorter, SCINA, Garnett, and CellAssign cannot be utilized to extract low-dimensional embeddings. Bottom, PAGA graphs generated by SpatialAnno, PCA, and DR-SC embeddings.

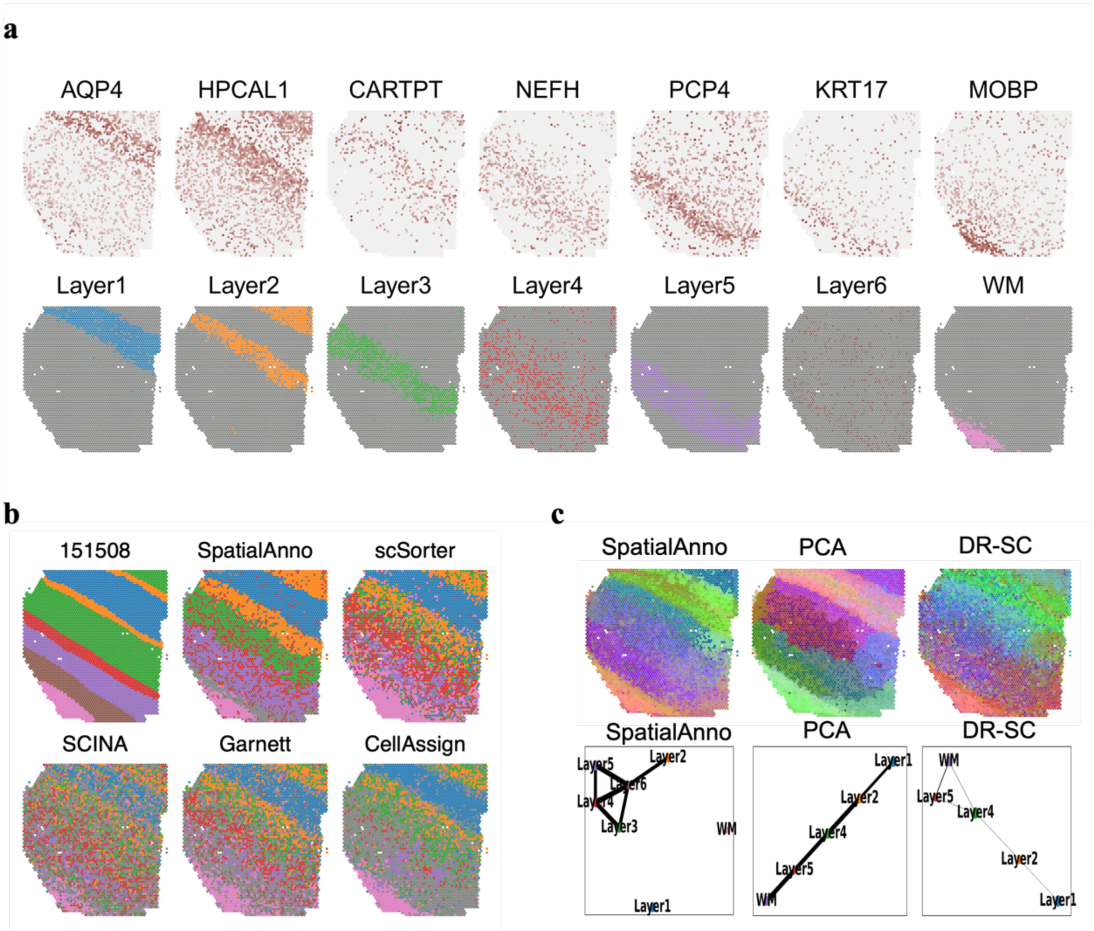

Supplementary Figure 6. Spatial domain annotation of DLPFC section 151509

a Spatial domain annotations of tissue section 151509 are shown for ground truth, SpatialAnno, scSorter, SCINA, Garnett, and CellAssign. **b** Top, annotation by SpatialAnno for each spot. Bottom, expression levels of corresponding layer-specific marker genes. **c** Top, RGB plots for low-dimensional embedding inferred by SpatialAnno, PCA, and DR-SC. As end-to-end annotation approaches, scSorter, SCINA, Garnett, and CellAssign cannot be utilized to extract low-dimensional embeddings. Bottom, PAGA graphs generated by SpatialAnno, PCA, and DR-SC embeddings.

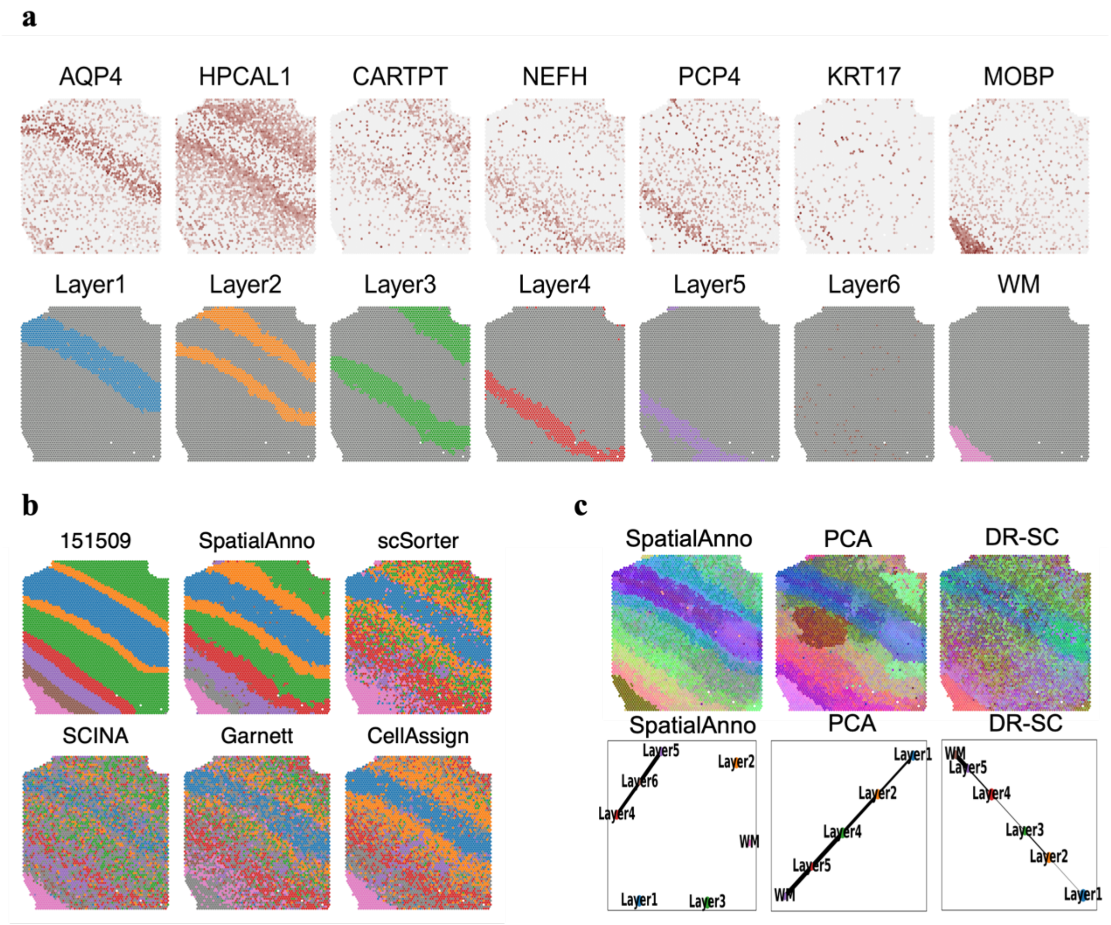

Supplementary Figure 7. Spatial domain annotation of DLPFC section 151510

a Spatial domain annotations of tissue section 151510 are shown for ground truth, SpatialAnno, scSorter, SCINA, Garnett, and CellAssign. **b** Top, annotation of SpatialAnno for each spot. Bottom, expression levels of corresponding layer-specific marker genes. **c** Top, RGB plots for low-dimensional embedding inferred by SpatialAnno, PCA, and DR-SC. As end-to-end annotation approaches, scSorter, SCINA, Garnett, and CellAssign cannot be utilized to extract low-dimensional embeddings. Bottom, PAGA graphs generated by SpatialAnno, PCA, and DR-SC embeddings.

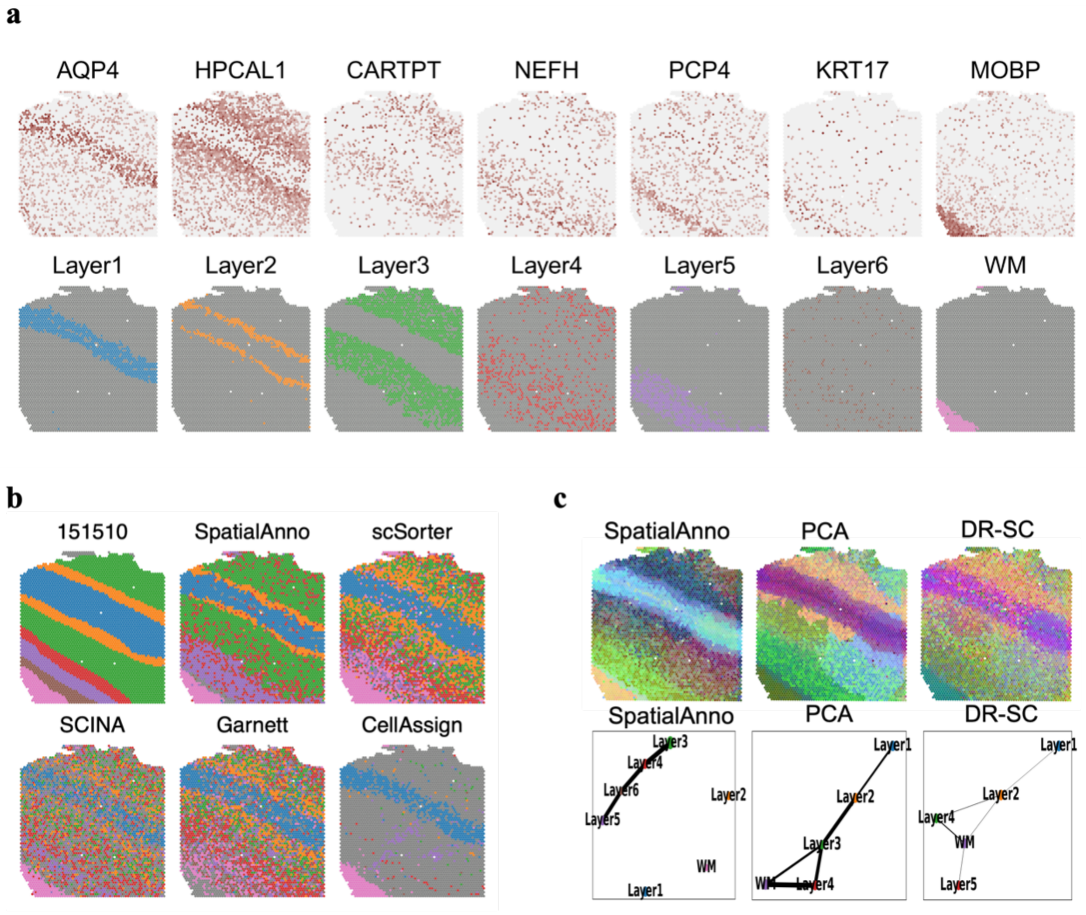

Supplementary Figure 8. Spatial domain annotation of DLPFC section 151669

a Spatial domain annotations of tissue section 151669 are shown for ground truth, SpatialAnno, scSorter, SCINA, Garnett, and CellAssign. **b** Top, annotation of SpatialAnno for each spot. Bottom, expression levels of corresponding layer-specific marker genes. **c** Top, RGB plots for low-dimensional embedding inferred by SpatialAnno, PCA, and DR-SC. As end-to-end annotation approaches, scSorter, SCINA, Garnett, and CellAssign cannot be utilized to extract low-dimensional embeddings. Bottom, PAGA graphs generated by SpatialAnno, PCA, and DR-SC embeddings.

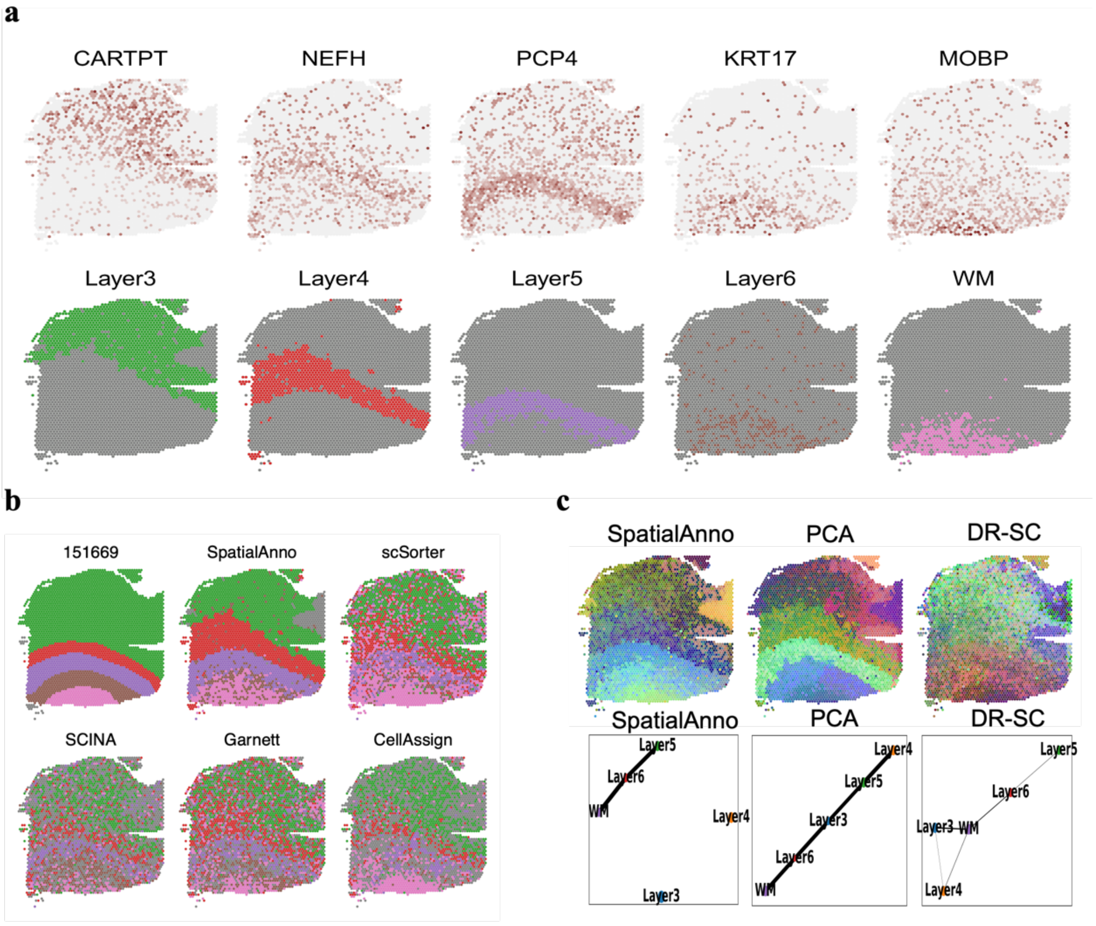

Supplementary Figure 9. Spatial domain annotation of DLPFC section 151670

a Spatial domain annotations of tissue section 151670 are shown for ground truth, SpatialAnno, scSorter, SCINA, Garnett, and CellAssign. **b** Top, annotation of SpatialAnno for each spot. Bottom, expression levels of corresponding layer-specific marker genes. **c** Top, RGB plots for low-dimensional embedding inferred by SpatialAnno, PCA, and DR-SC. As end-to-end annotation approaches, scSorter, SCINA, Garnett, and CellAssign cannot be utilized to extract low-dimensional embeddings. Bottom, PAGA graphs generated by SpatialAnno, PCA, and DR-SC embeddings.

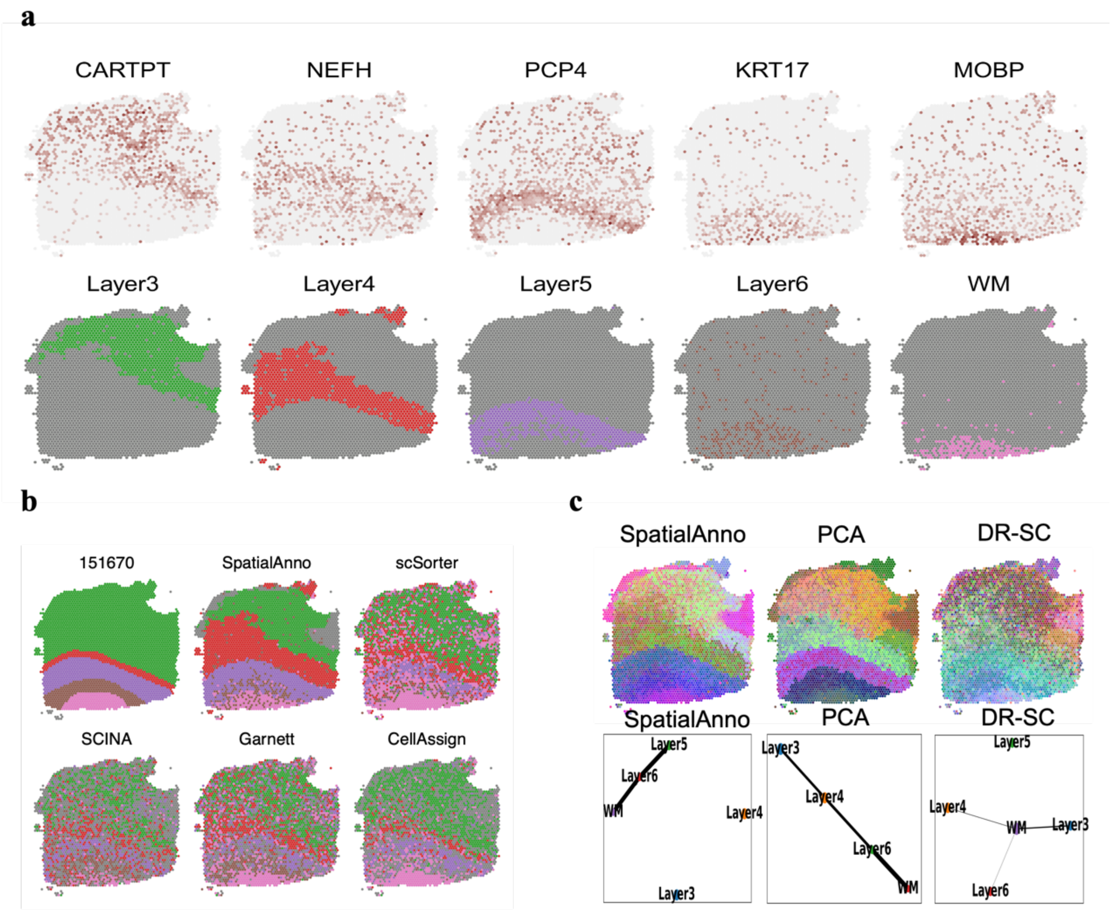

Supplementary Figure 10. Spatial domain annotation of DLPFC section 151671
a Spatial domain annotations of tissue section 151671 are shown for ground truth, SpatialAnno, scSorter, SCINA, Garnett, and CellAssign. **b** Top, annotation of SpatialAnno for each spot. Bottom, expression levels of corresponding layer-specific marker genes. **c** Top, RGB plots for low-dimensional embedding inferred by SpatialAnno, PCA, and DR-SC. As end-to-end annotation approaches, scSorter, SCINA, Garnett, and CellAssign cannot be utilized to extract low-dimensional embeddings. Bottom, PAGA graphs generated by SpatialAnno, PCA, and DR-SC embeddings.

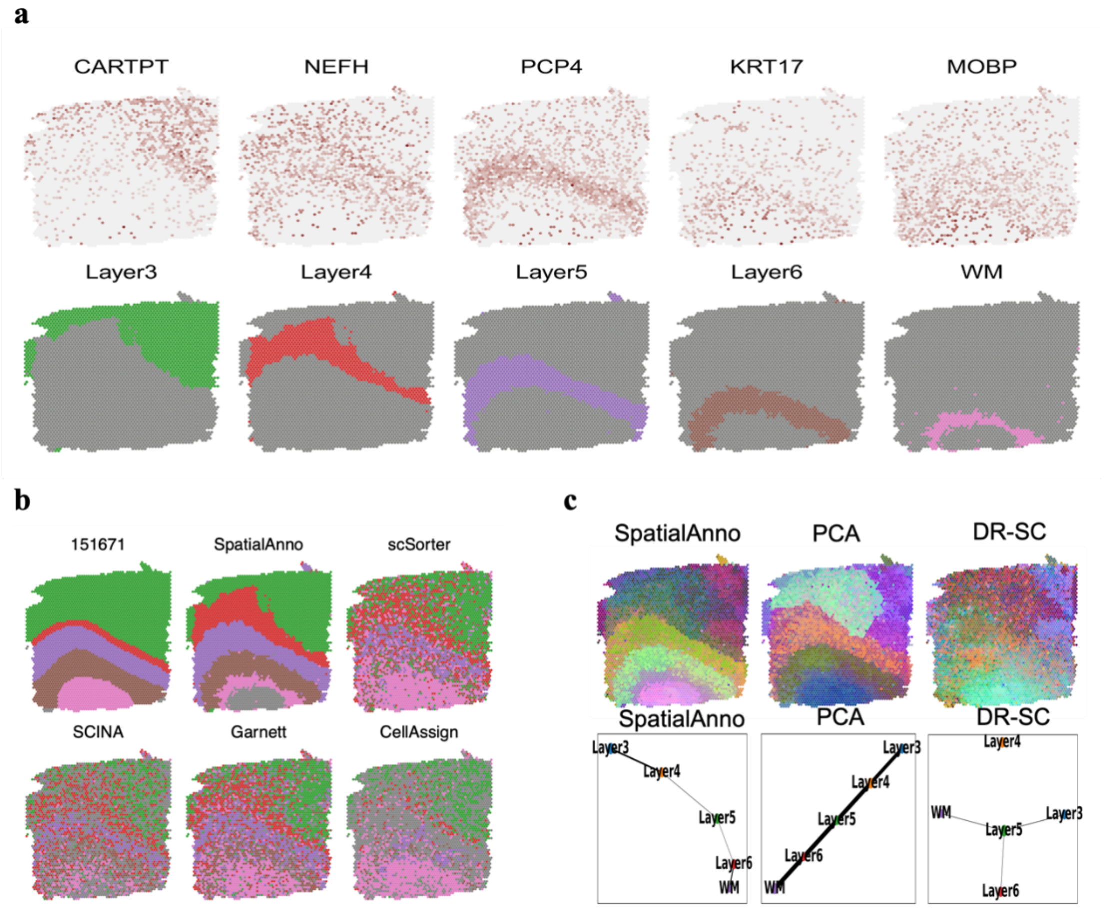

Supplementary Figure 11. Spatial domain annotation of DLPFC section 151672

a Spatial domain annotation of tissue section 151672 are shown for ground truth, SpatialAnno, scSorter, SCINA, Garnett, and CellAssign. **b** Top, annotation of SpatialAnno for each spot. Bottom, expression levels of corresponding layer-specific marker genes. **c** Top, RGB plots for low-dimensional embedding inferred by SpatialAnno, PCA, and DR-SC. As end-to-end annotation approaches, scSorter, SCINA, Garnett, and CellAssign cannot be utilized to extract low-dimensional embeddings. Bottom, PAGA graphs generated by SpatialAnno, PCA, and DR-SC embeddings.

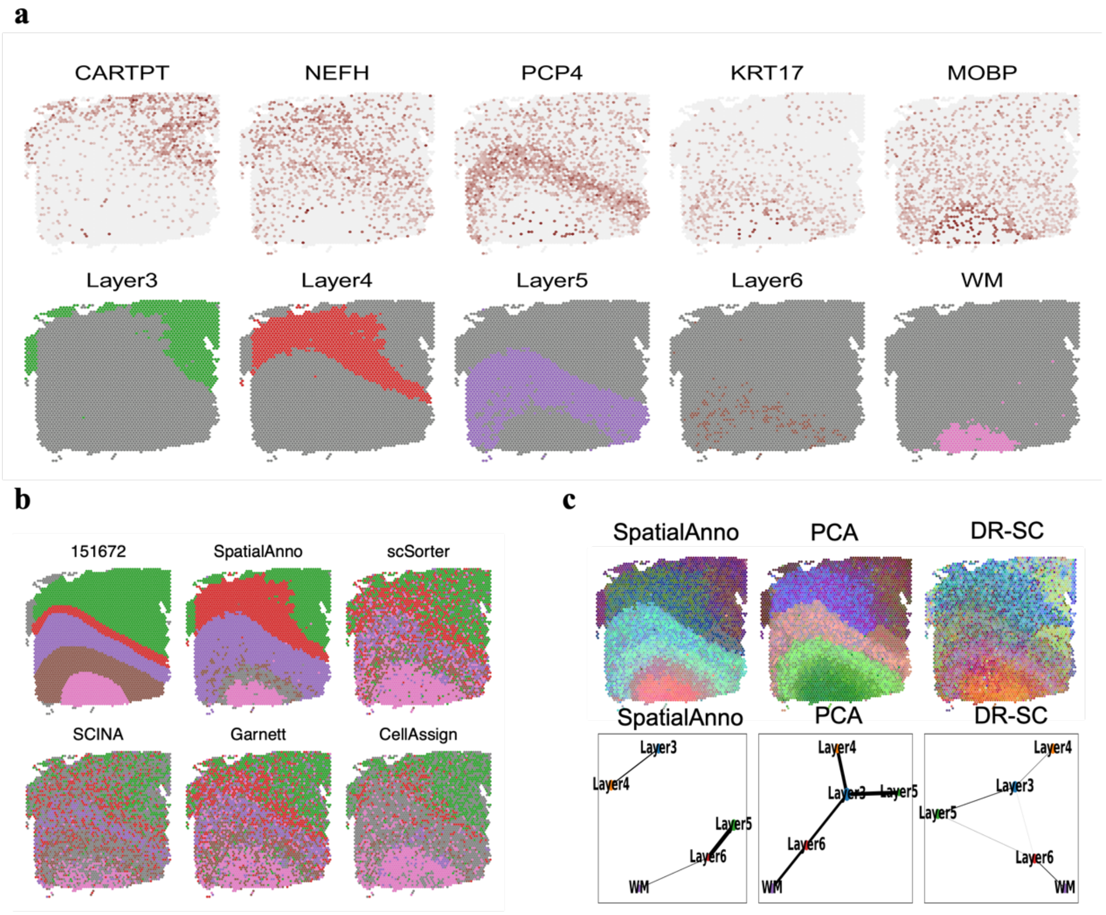

Supplementary Figure 12. Spatial domain annotation of DLPFC section 151673
a Spatial domain annotations of tissue section 151673 are shown for ground truth, SpatialAnno, scSorter, SCINA, Garnett, and CellAssign. **b** Top, annotation by SpatialAnno for each spot. Bottom, expression levels of corresponding layer-specific marker genes. **c** Top, RGB plots for low-dimensional embedding inferred by SpatialAnno, PCA, and DR-SC. As end-to-end annotation approaches, scSorter, SCINA, Garnett, and CellAssign cannot be utilized to extract low-dimensional embeddings. Bottom, PAGA graphs generated by SpatialAnno, PCA, and DR-SC embeddings.

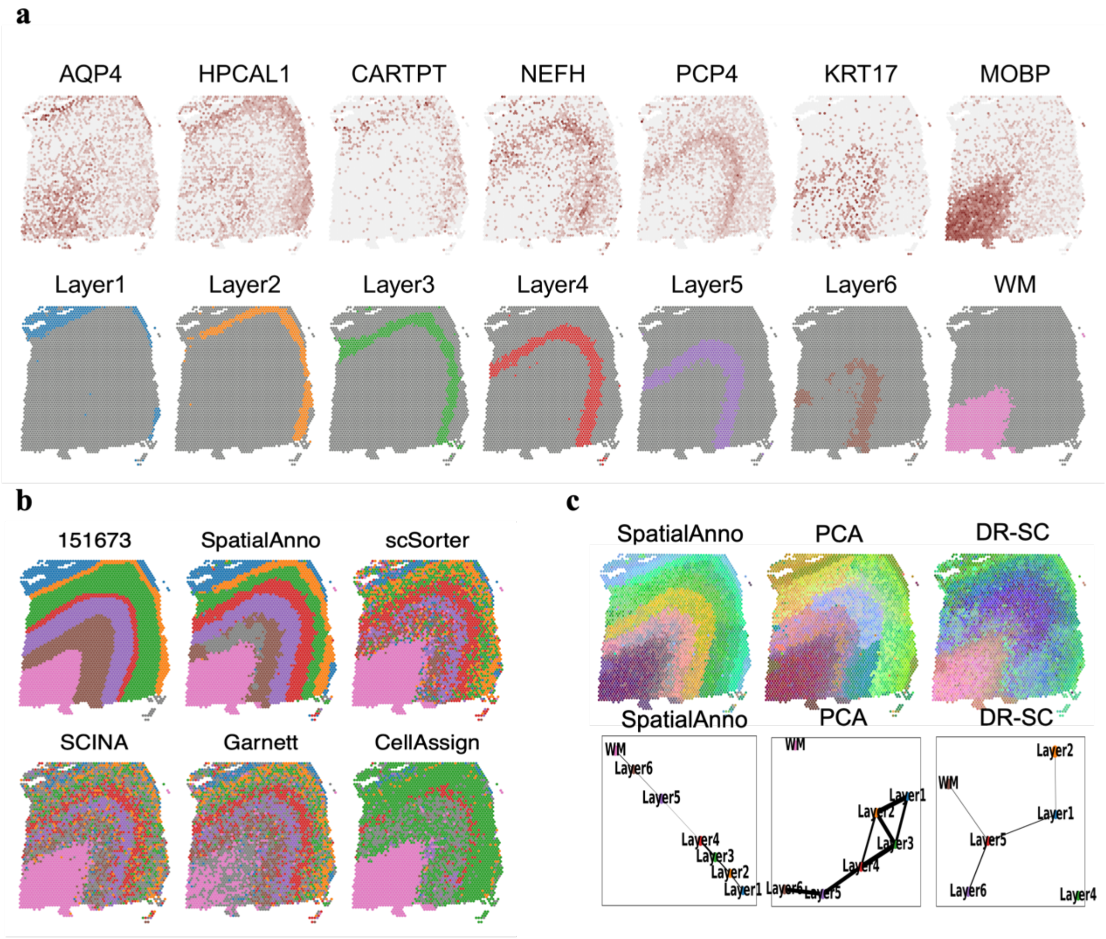

Supplementary Figure 13. Spatial domain annotation of DLPFC section 151674

a Spatial domain annotation of tissue section 151674 are shown for ground truth, SpatialAnno, scSorter, SCINA, Garnett, and CellAssign. **b** Top, annotation by SpatialAnno for each spot. Bottom, expression levels of corresponding layer-specific marker genes. **c** Top, RGB plots for low-dimensional embedding inferred by SpatialAnno, PCA, and DR-SC. As end-to-end annotation approaches, scSorter, SCINA, Garnett, and CellAssign cannot be utilized to extract low-dimensional embeddings. Bottom, PAGA graphs generated by SpatialAnno, PCA, and DR-SC embeddings.

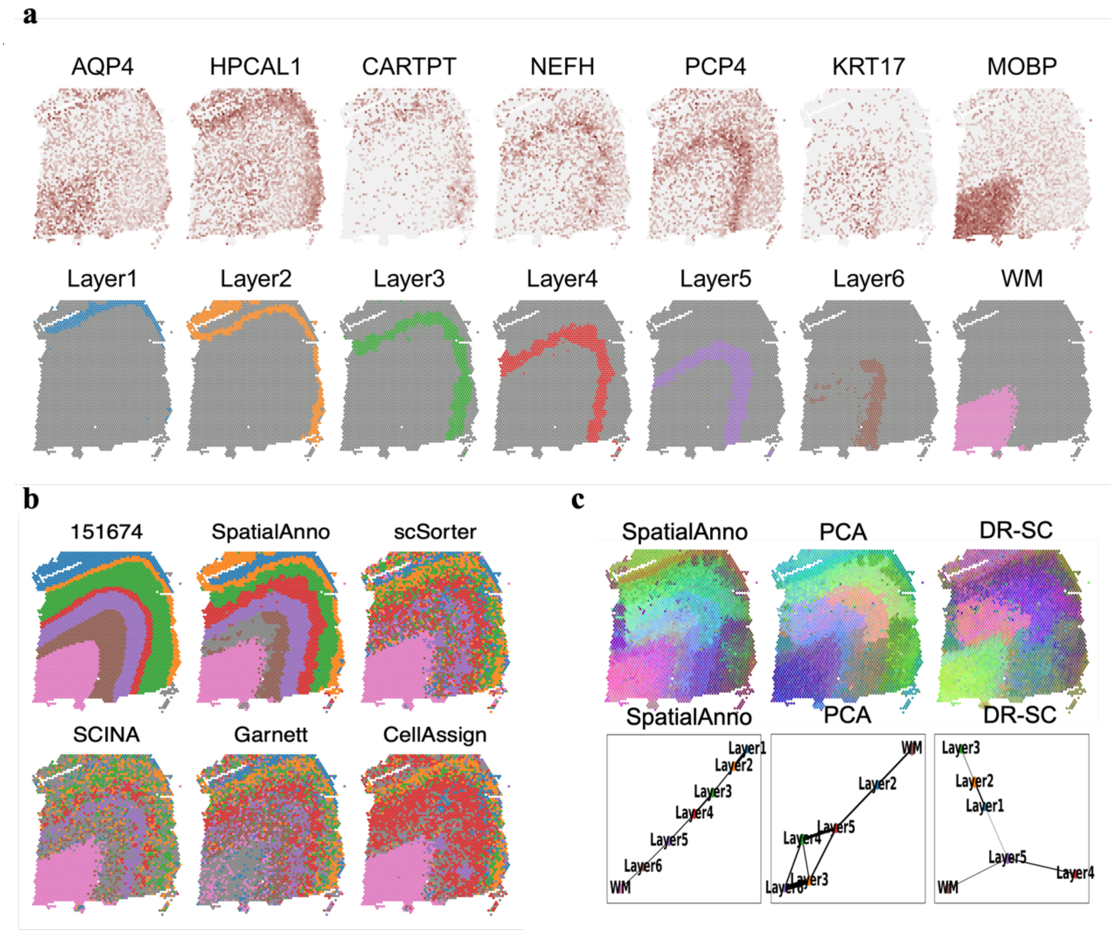

Supplementary Figure 14. Spatial domain annotation of DLPFC section 151675
a Spatial domain annotation of tissue section 151675 are shown for ground truth, SpatialAnno, scSorter, SCINA, Garnett, and CellAssign. **b** Top, annotation of SpatialAnno for each spot. Bottom, expression levels of corresponding layer-specific marker genes. **c** Top, RGB plots for low-dimensional embedding inferred by SpatialAnno, PCA, and DR-SC. As end-to-end annotation approaches, scSorter, SCINA, Garnett, and CellAssign cannot be utilized to extract low-dimensional embeddings. Bottom, PAGA graphs generated by SpatialAnno, PCA, and DR-SC embeddings.

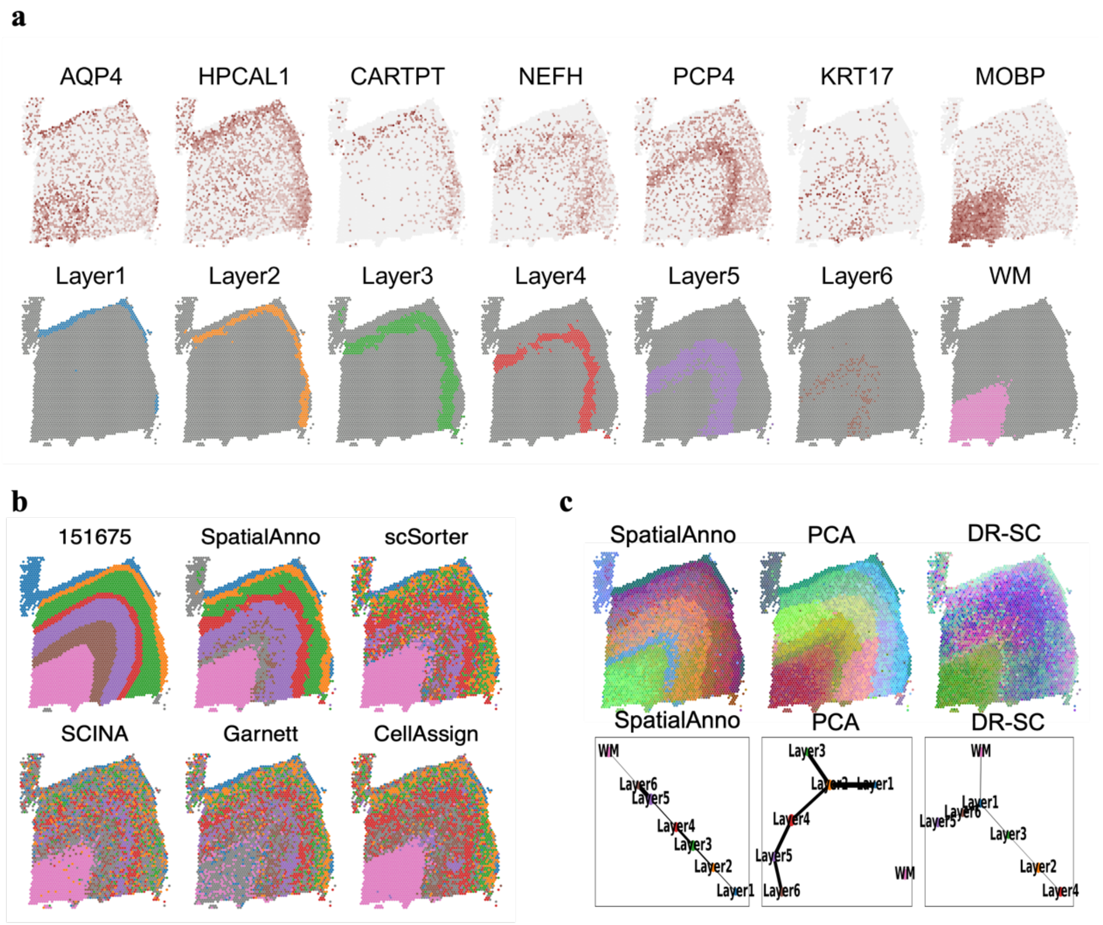

Supplementary Figure 15. Spatial domain annotation of DLPFC Section 151676
a Spatial domain annotation of tissue section 151676 are shown for ground truth, SpatialAnno, scSorter, SCINA, Garnett, and CellAssign. **b** Top, annotation by SpatialAnno for each spot. Bottom, expression levels of corresponding layer-specific marker genes. **c** Top, RGB plots for low-dimensional embedding inferred by SpatialAnno, PCA, and DR-SC. As end-to-end annotation approaches, scSorter, SCINA, Garnett, and CellAssign cannot be utilized to extract low-dimensional embeddings. Bottom, PAGA graphs generated by SpatialAnno, PCA, and DR-SC embeddings.

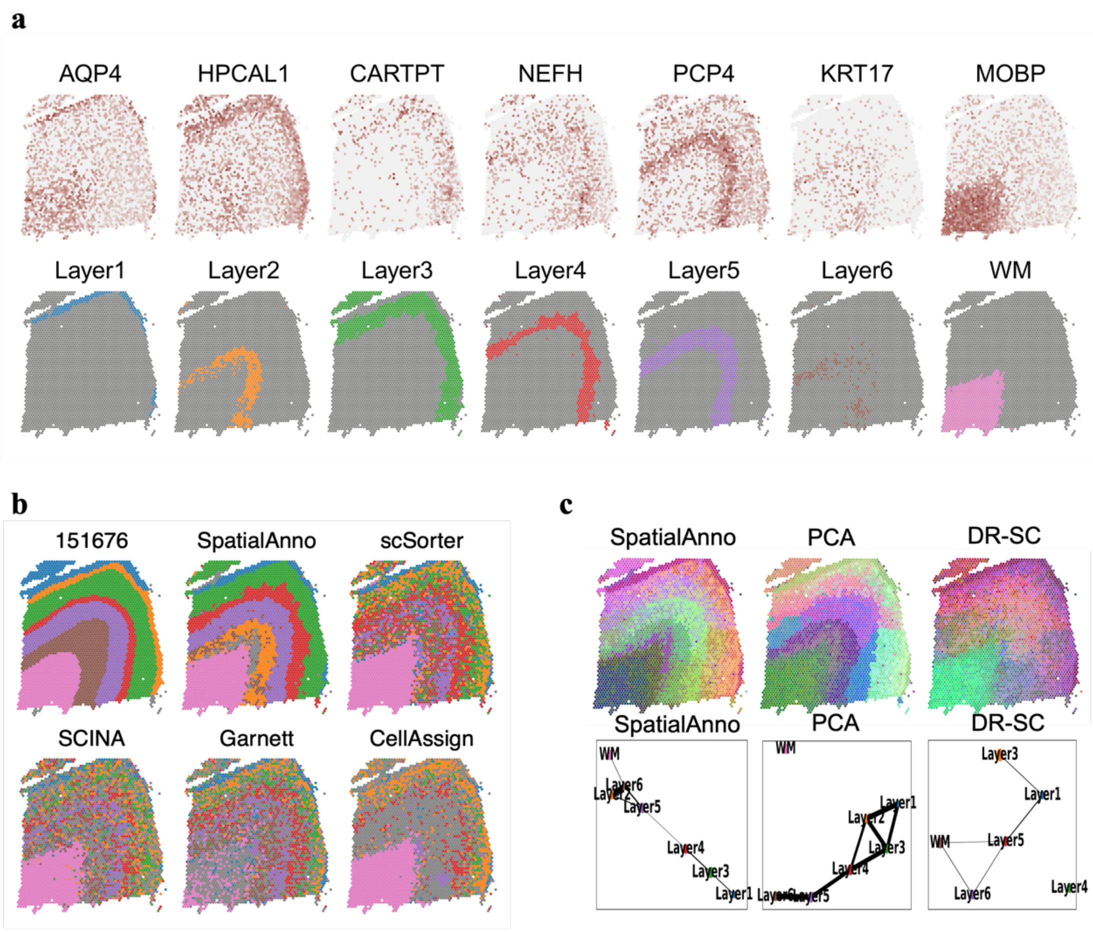

Supplementary Figure 16. Additional analysis results for the DLPFC 10x Visium dataset

a Boxplots for Kappa, mF1, and ACC show the accuracy of different methods using the top 5, 10, and 15 differentially expressed genes for domain annotation across 12 tissue sections. Two-sided Wilcoxon Rank Sum test was used to pair wisely test the difference between metrices of different methods, and the *p*-value is shown. **b** Boxplots of Kappa, mF1, and ACC show the accuracy of different methods when performing annotation with marker genes identified from section 151607 for samples with ID151669-151672 from Donor 2 that only contained five cortical layers. Correctly- (5) or over-specified (7) cell/domain types are provided in the marker gene list. **c** Clustering results measured by ARI (the higher the better) based on low-dimensional embeddings either from marker genes by PCA or non-marker genes by SpatialAnno, or a combination. Two-sided Wilcoxon Rank Sum test was used to pair wisely test the ARI difference, and the *p*-value is shown. **d** Boxplots for Kappa, mF1, and ACC show the performance of different components in SpaitalAnno model. Full: SpatialAnno; Marker+Nonmarker: SpatialAnno without spatial information; Spatial+Marker: SpatialAnno without nonmarker genes; Marker: SpatialAnno without nonmarker genes and spatial information.

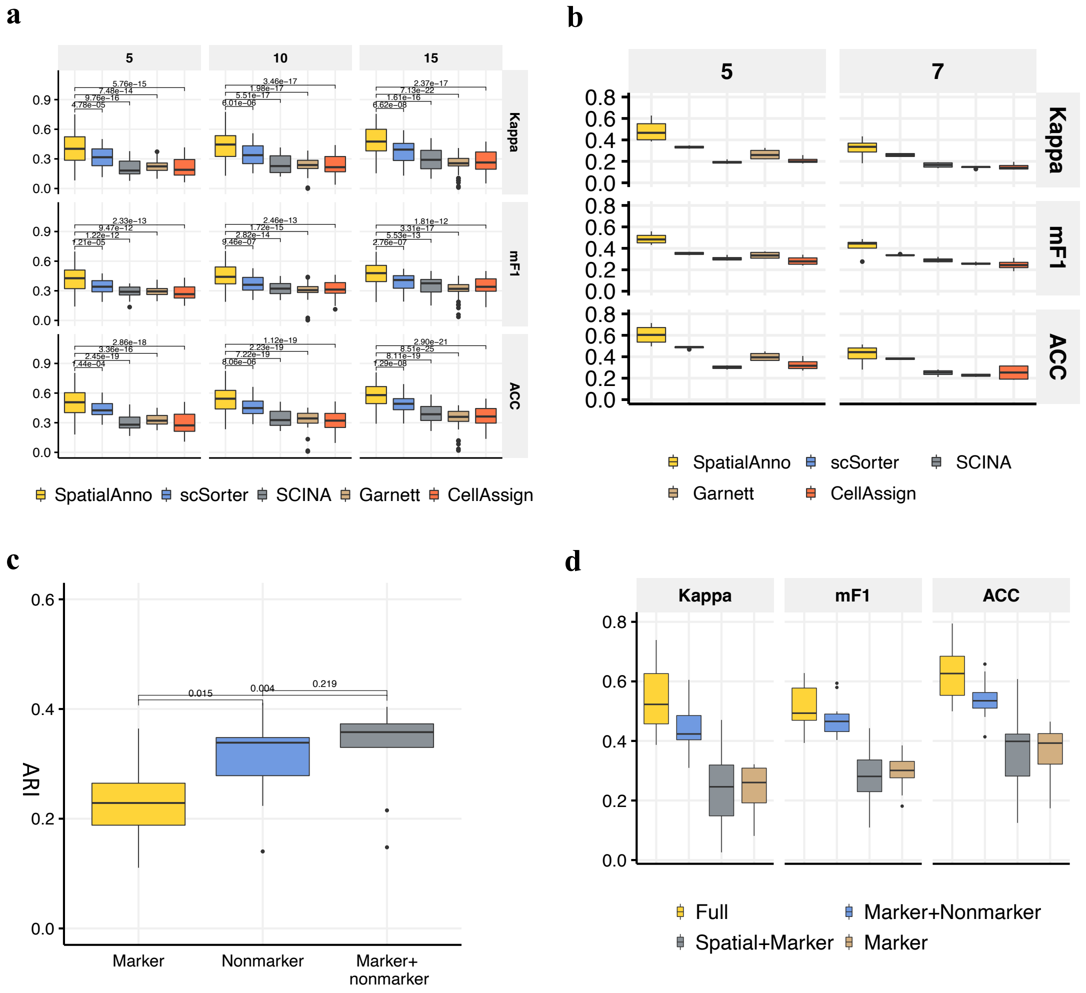

Supplementary Figure 17. Spatial annotation of mouse olfactory bulb dataset

a Bar plots of Kappa, mF1, and ACC showing the accuracy of the different methods when performing annotation with correctly- (5) or over-specified (7) cell types in the marker gene list. **b** Bar plots of Kappa and mF1 and ACC showing the accuracy of SpatialAnno, scSorter, and Garnett using 30, 300, and 3000 non-marker genes. **C** Bar plots of Kappa, mF1, and ACC showing the accuracy of different methods by manually combining EPL-IN and PGC. **d** Bar plots of Kappa, mF1, and ACC showing the accuracy of different methods by manually combing EPL-IN, M/TC, and PGC. **e** tSNE plot for SpatialAnno, where tSNE PCs were obtained based on the extracted 15-dimensional SpatialAnno embeddings.

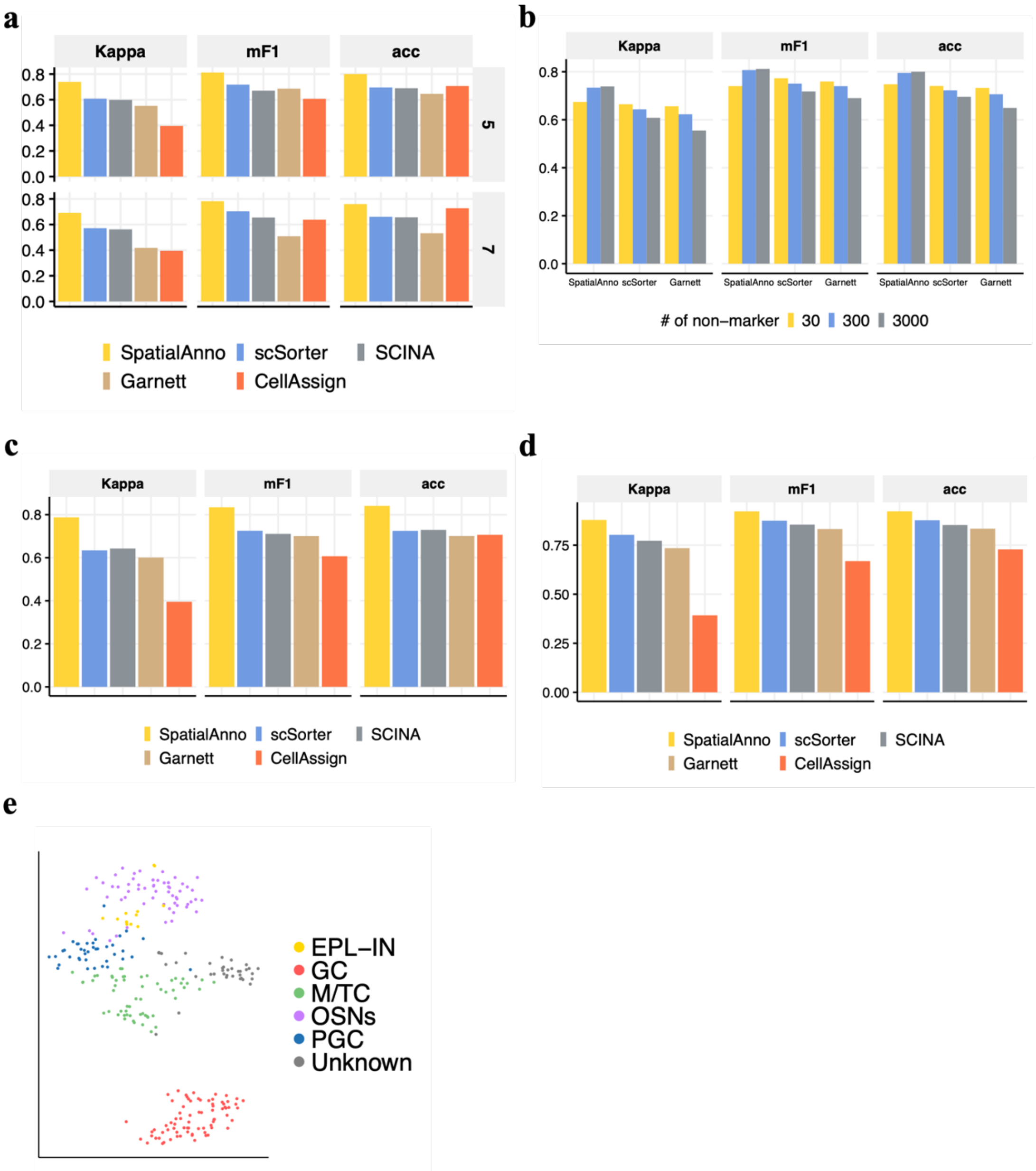

Supplementary Figure 18. Spatial distribution of cell types in the mouse olfactory bulb dataset across 12 sections annotated via different methods

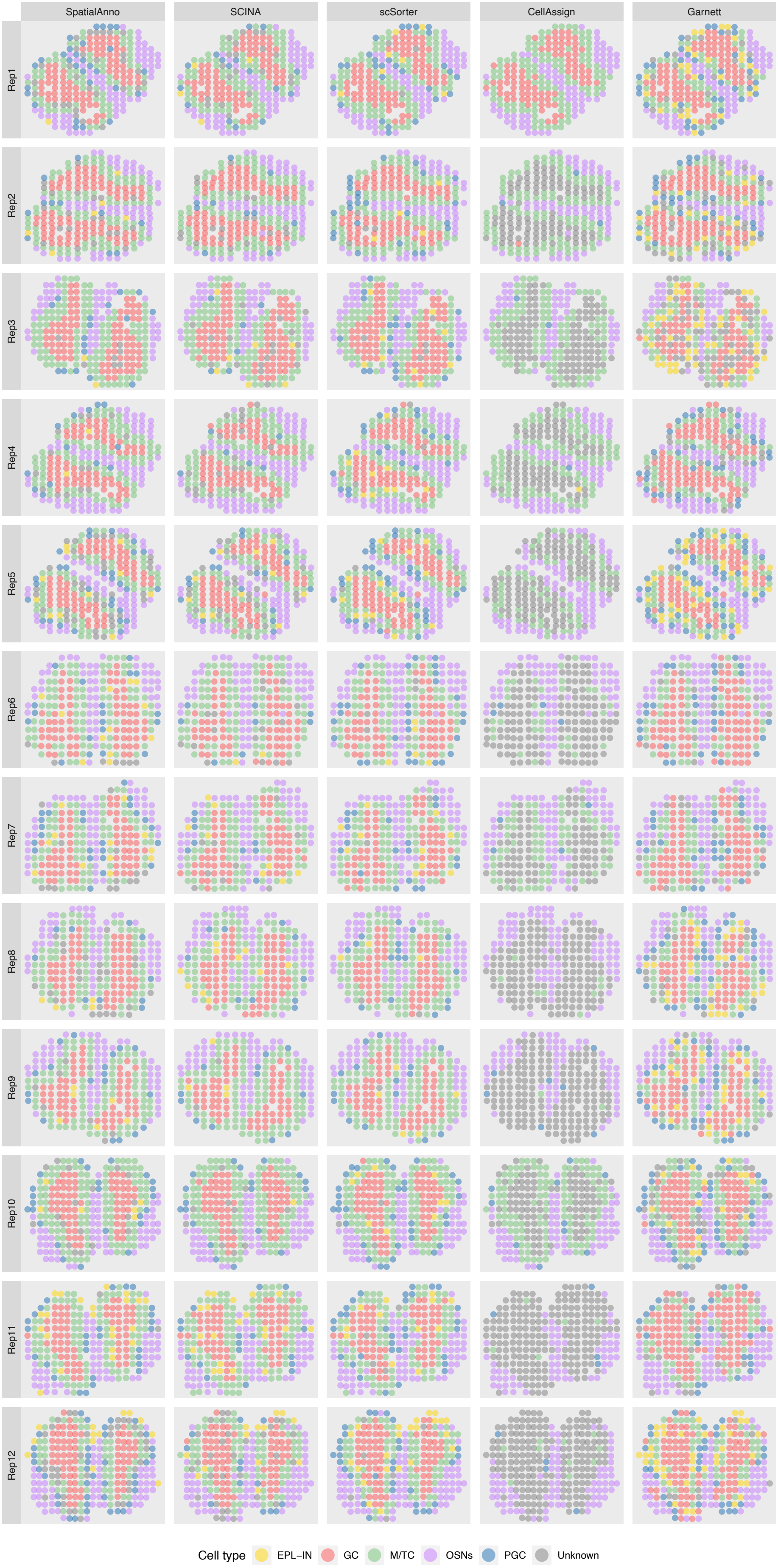

Supplementary Figure 19. Spatial domain annotations in mouse olfactory bulb section 12 by SpatialAnno, scSorter, SCINA, Garnett, and CellAssign

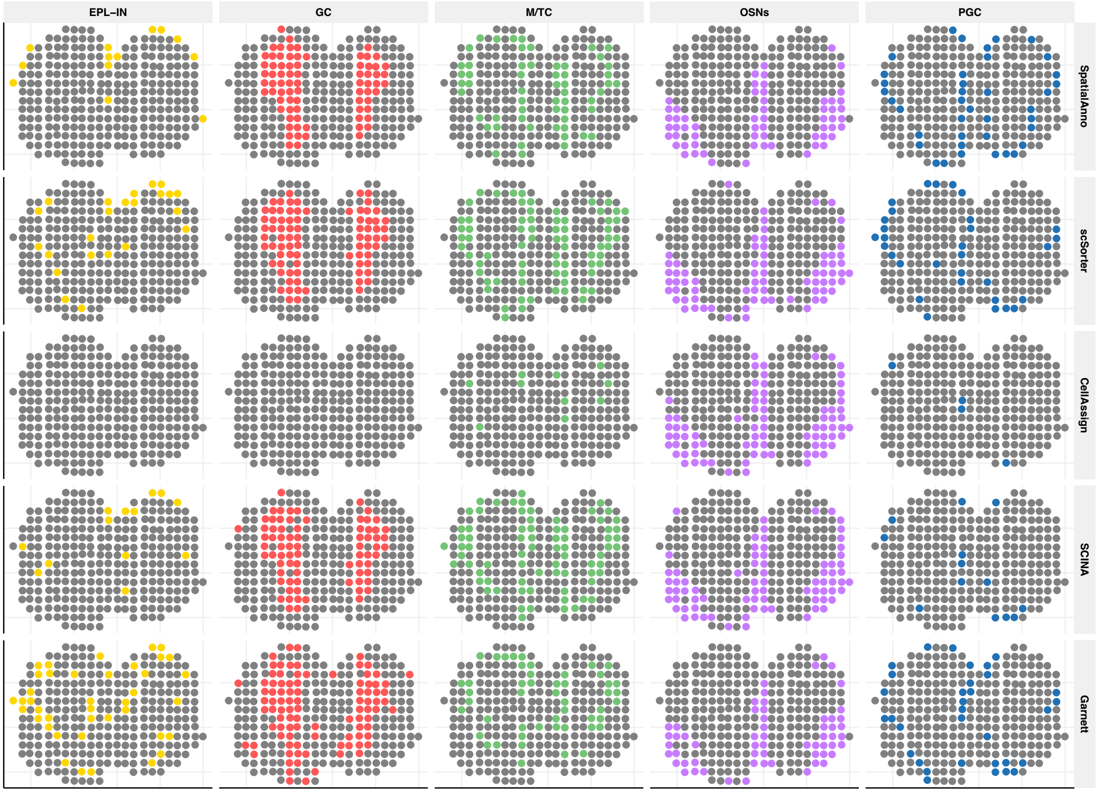

Supplementary Figure 20. Spatial distribution of cell types in the mouse hippocampus Slide-seqV2 data annotated by different methods

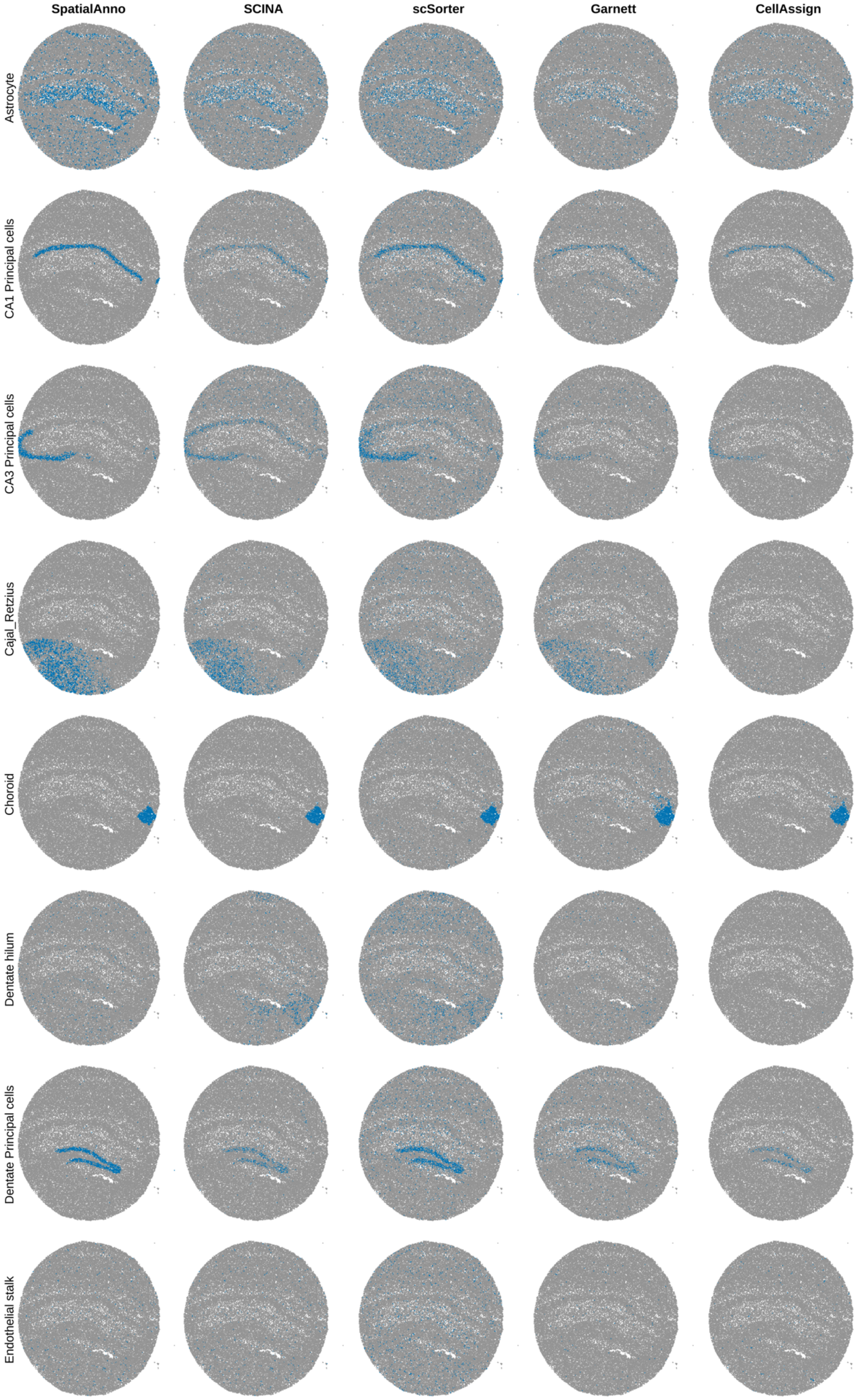

Supplementary Figure 21. Spatial distribution of cell types in the mouse hippocampus Slide-seqV2 data annotated by different methods (continued)

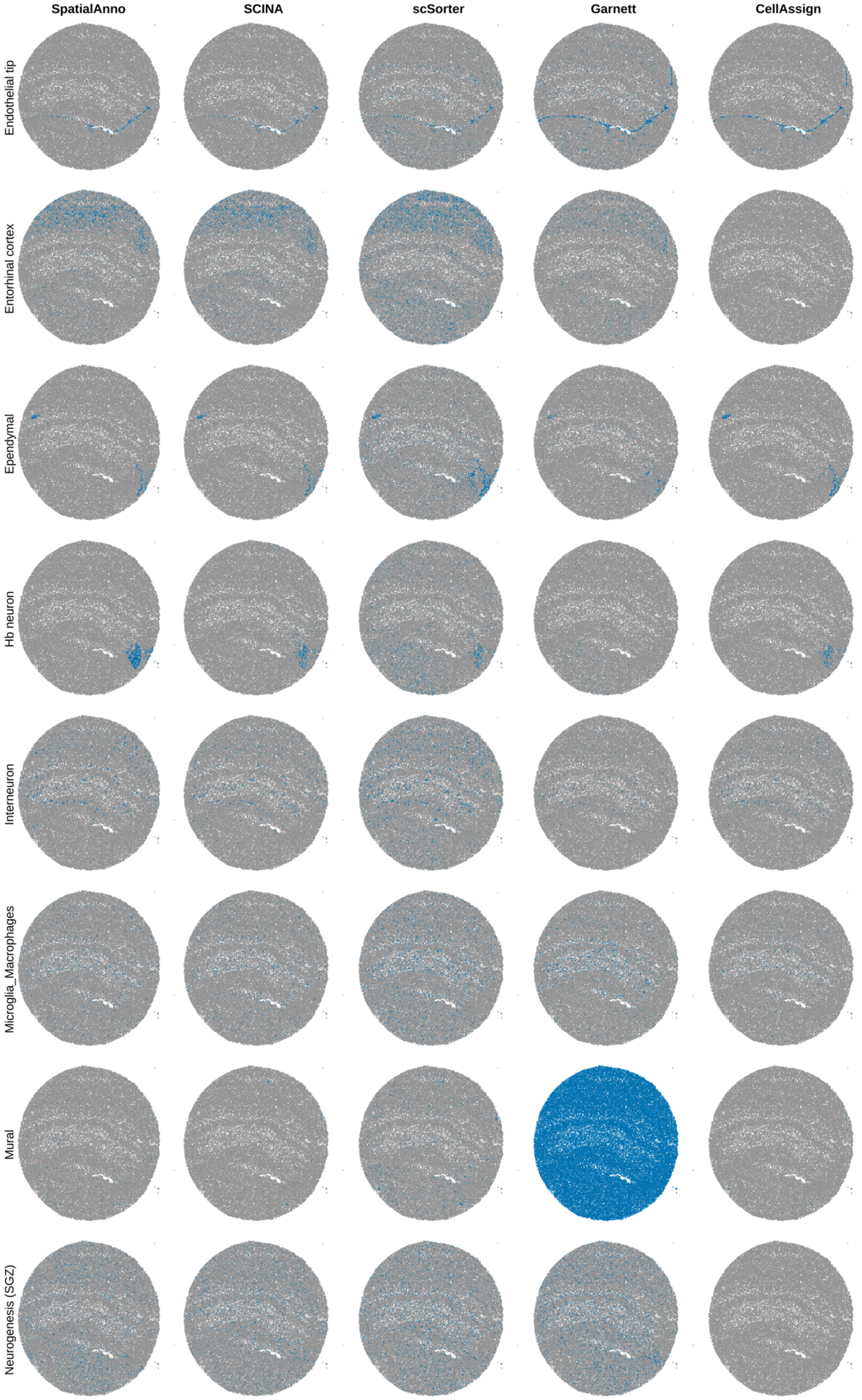

Supplementary Figure 22. Spatial distribution of cell types in the mouse hippocampus Slide-seqV2 data annotated by different methods (continued)

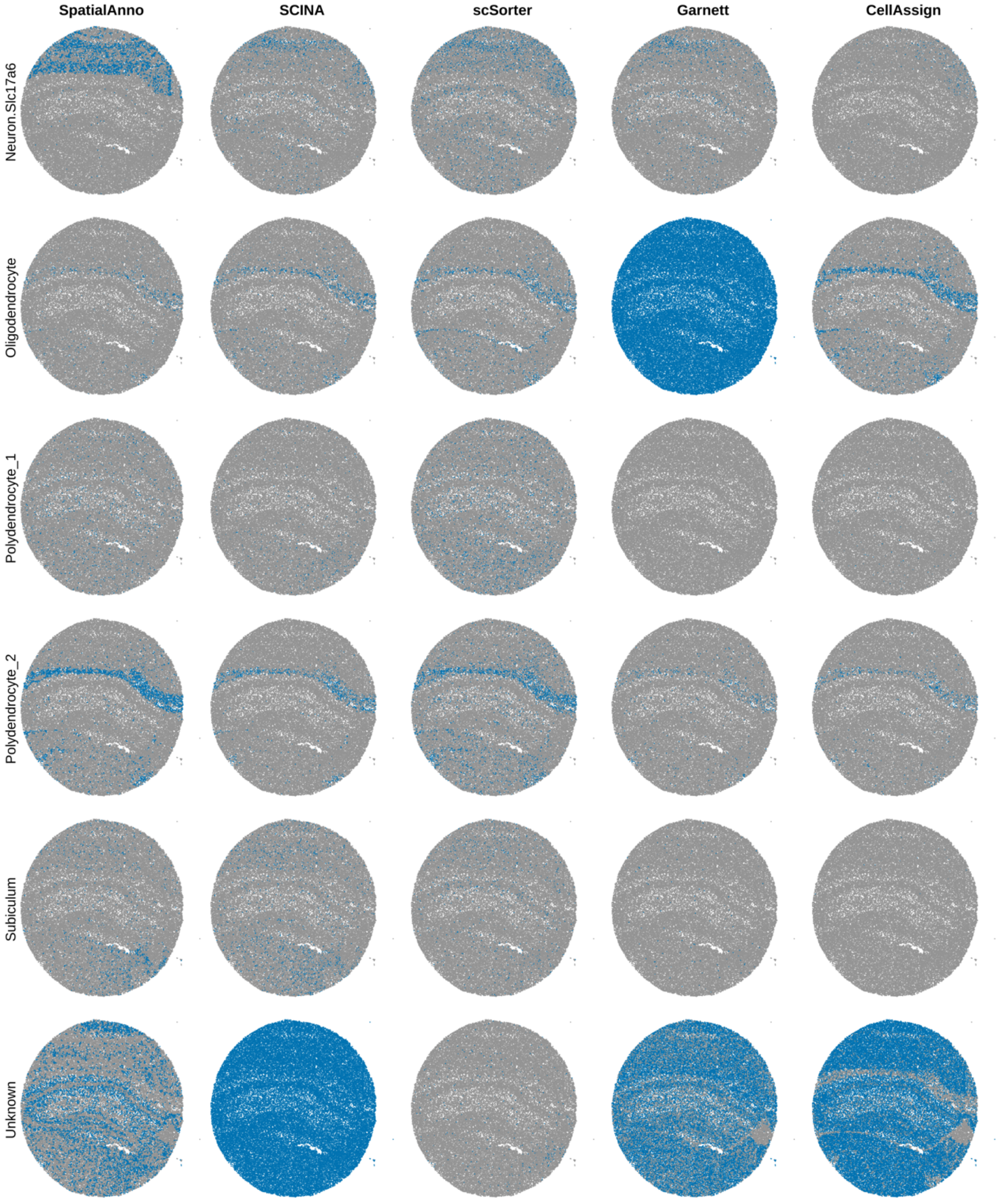

Supplementary Figure 23. Visualizations and annotations of mouse hippocampus Slide-seqV2 and Slide-seq data

a For SpatialAnno, PCA, and DR-SC, the inferred low-dimensional components of Slide-seqV2 data were summarized into three tSNE components and visualized with RGB plots. **b** Spatial annotation of Slide-seq data for SpatialAnno, scSorter, SCINA, Garnett, and CellAssign**.** **c** Results of Pearson's chi-squared test of correlation between the expression patterns of marker genes and the three hippocampal subfields identified by different methods in Slide-seq data. **d** For SpatialAnno, PCA, and DR-SC, the inferred low-dimensional components of Slide-seq data were summarized into three tSNE components and visualized with RGB plots.

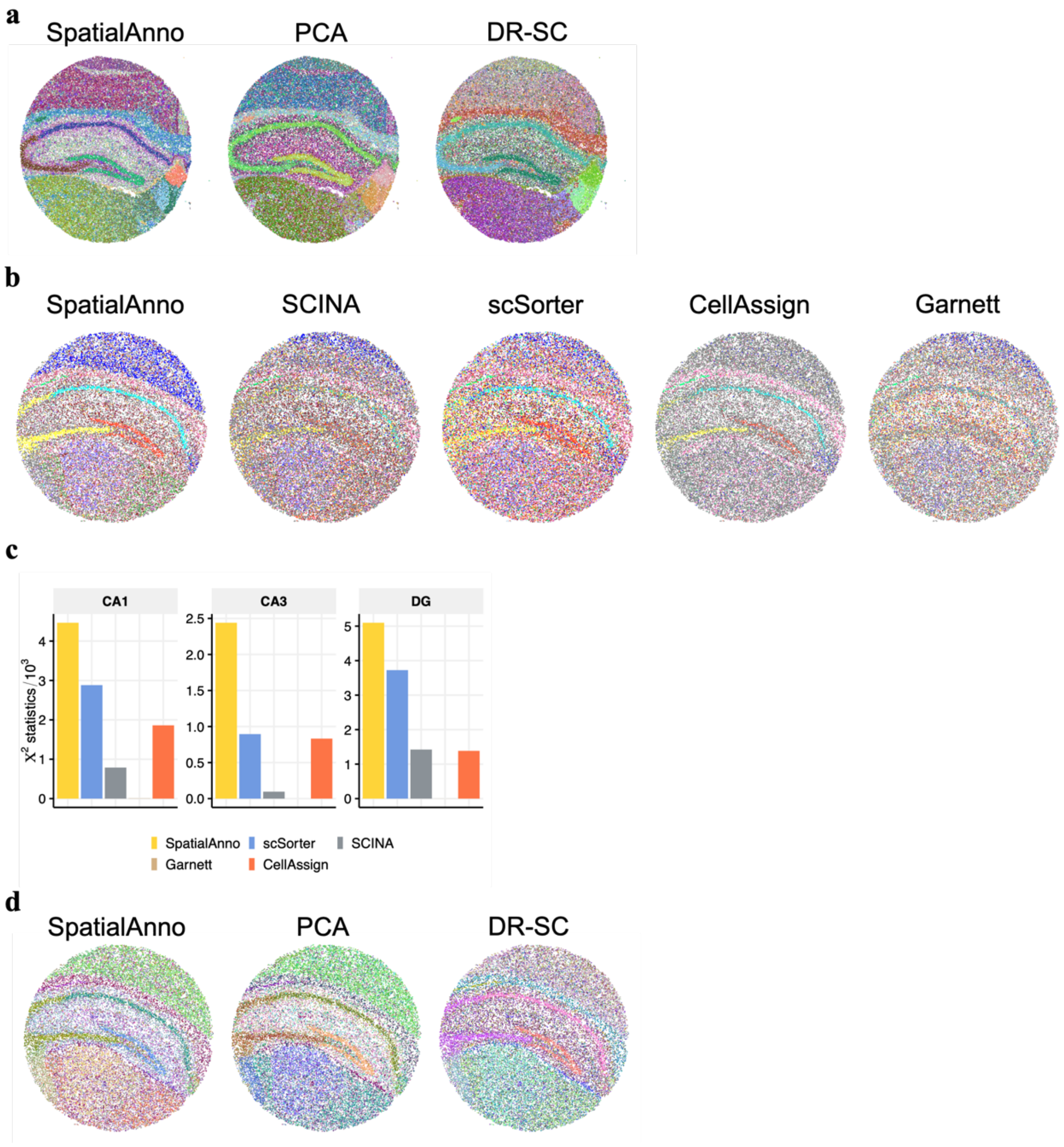

Supplementary Figure 24. Spatial distribution of cell types in the mouse hippocampus Slide-seqV1 data annotated by different methods

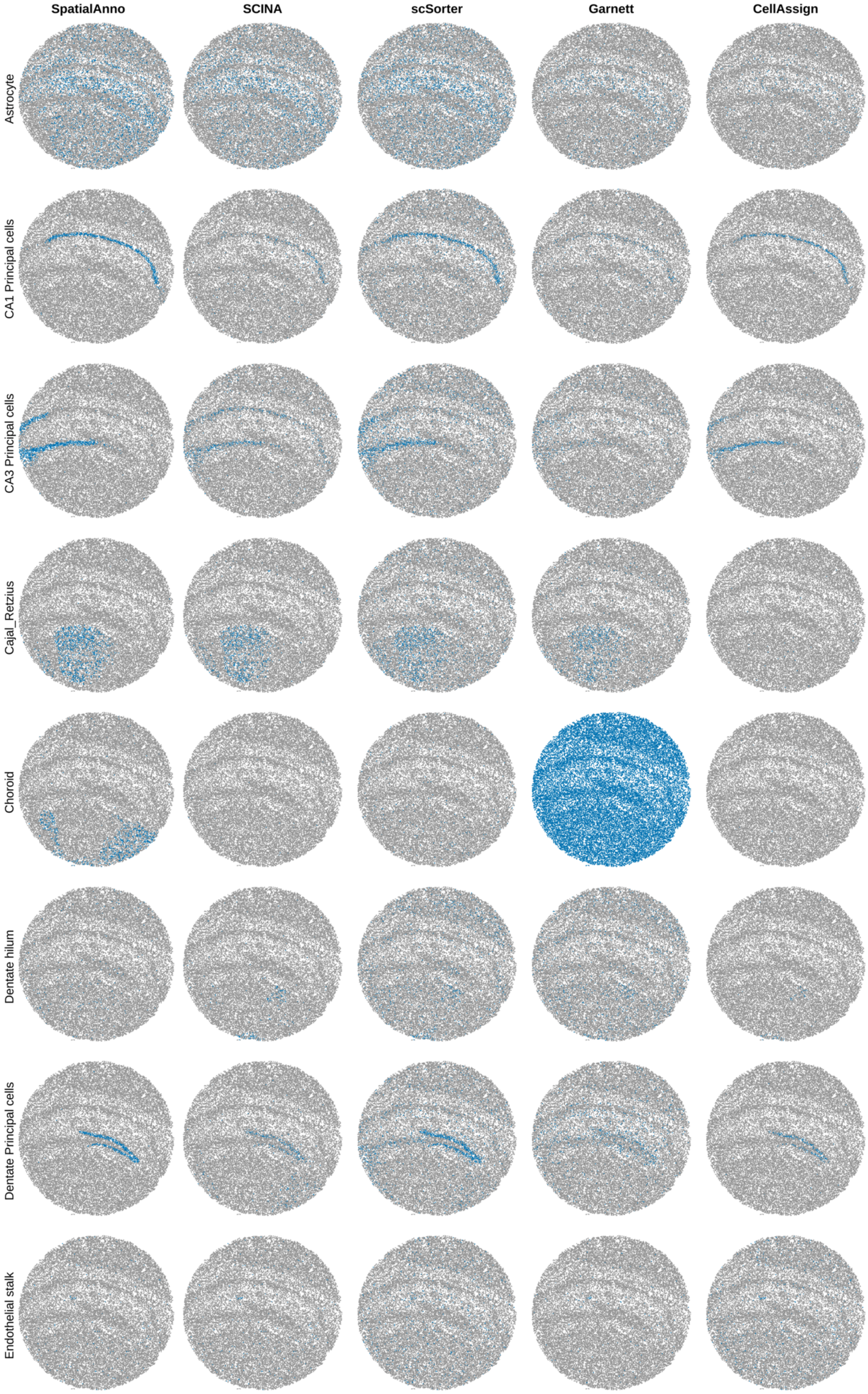

Supplementary Figure 25. Spatial distribution of cell types in the mouse hippocampus Slide-seqV1 data annotated by different methods (continued)

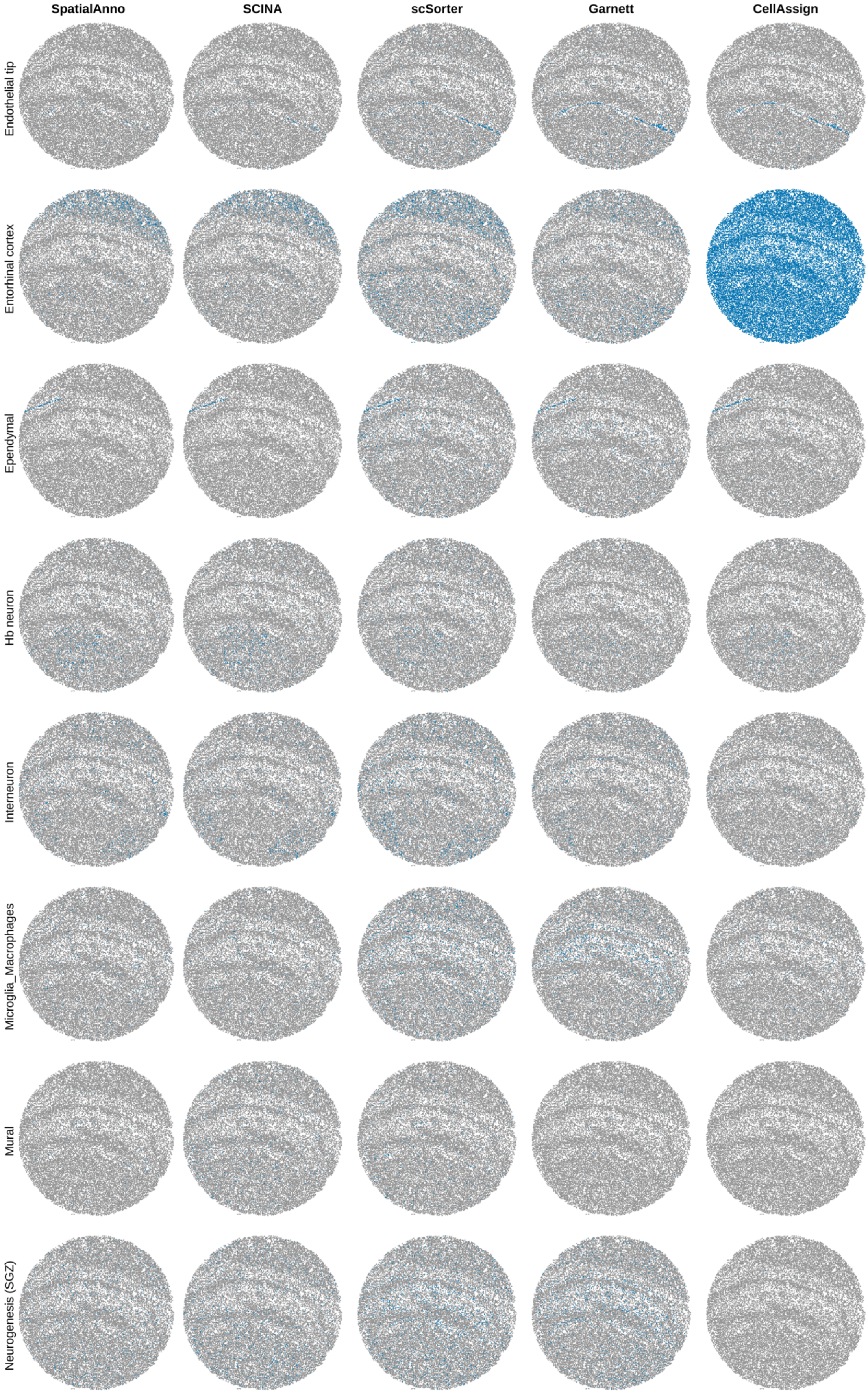

Supplementary Figure 26. Spatial distribution of cell types in the mouse hippocampus Slide-seqV1 data annotated by different methods (continued)

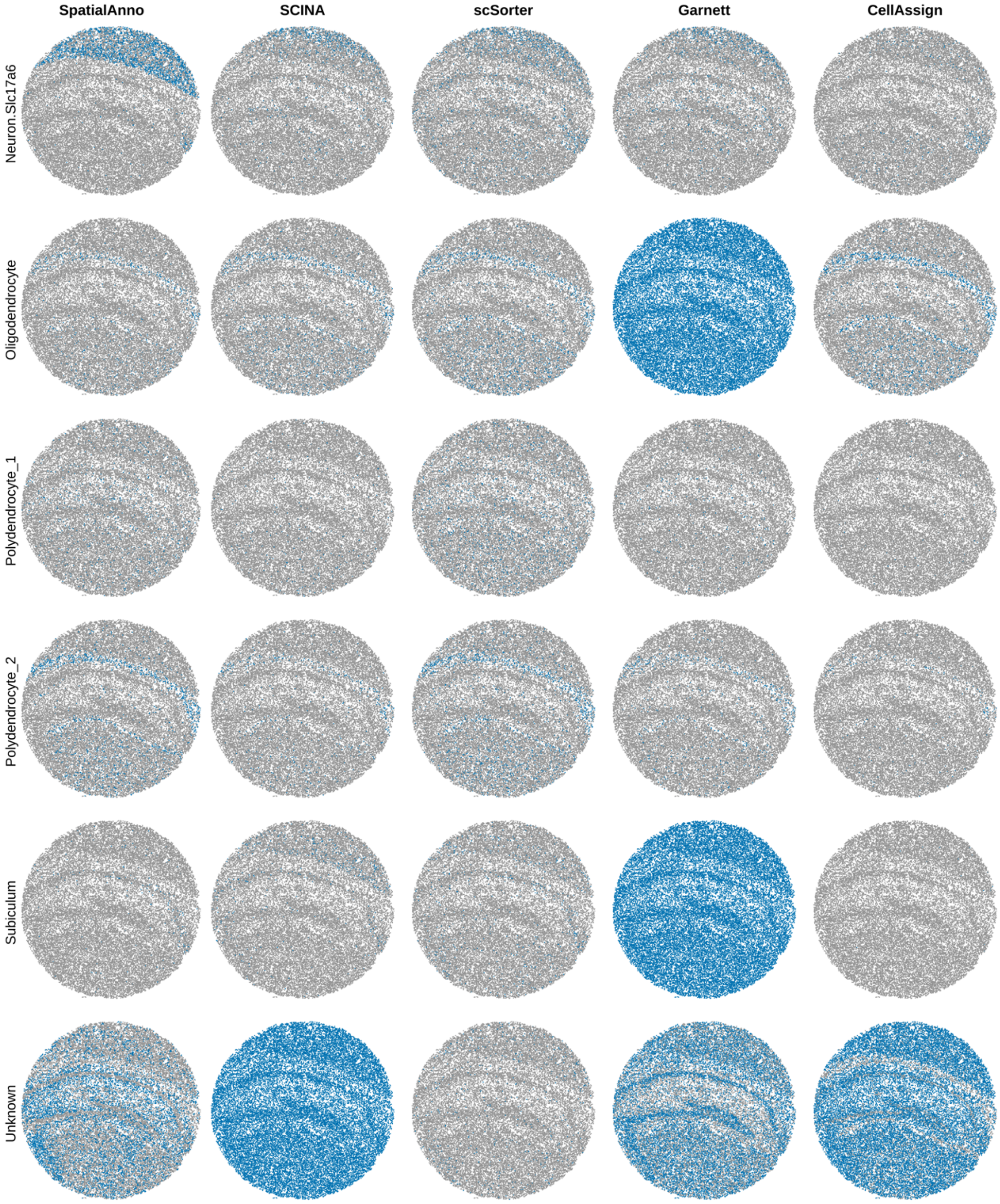

Supplementary Figure 27. Annotation results for mouse embryo 2 and 3 from seqFISH

a Bar plots of Kappa, mF1, and ACC showing the accuracy of different methods for cell type annotation of embryo 2. **b** Spatial annotations are shown for ground truth, SpatialAnno, scSorter, SCINA, Garnett, and CellAssign in embryo 2. **c** Bar plots of Kappa, mF1 and ACC showing the accuracy of different methods for cell type annotation of embryo 3. **d** Spatial annotations in embryo 3 are shown for ground truth, SpatialAnno, scSorter, SCINA, Garnett, and CellAssign.

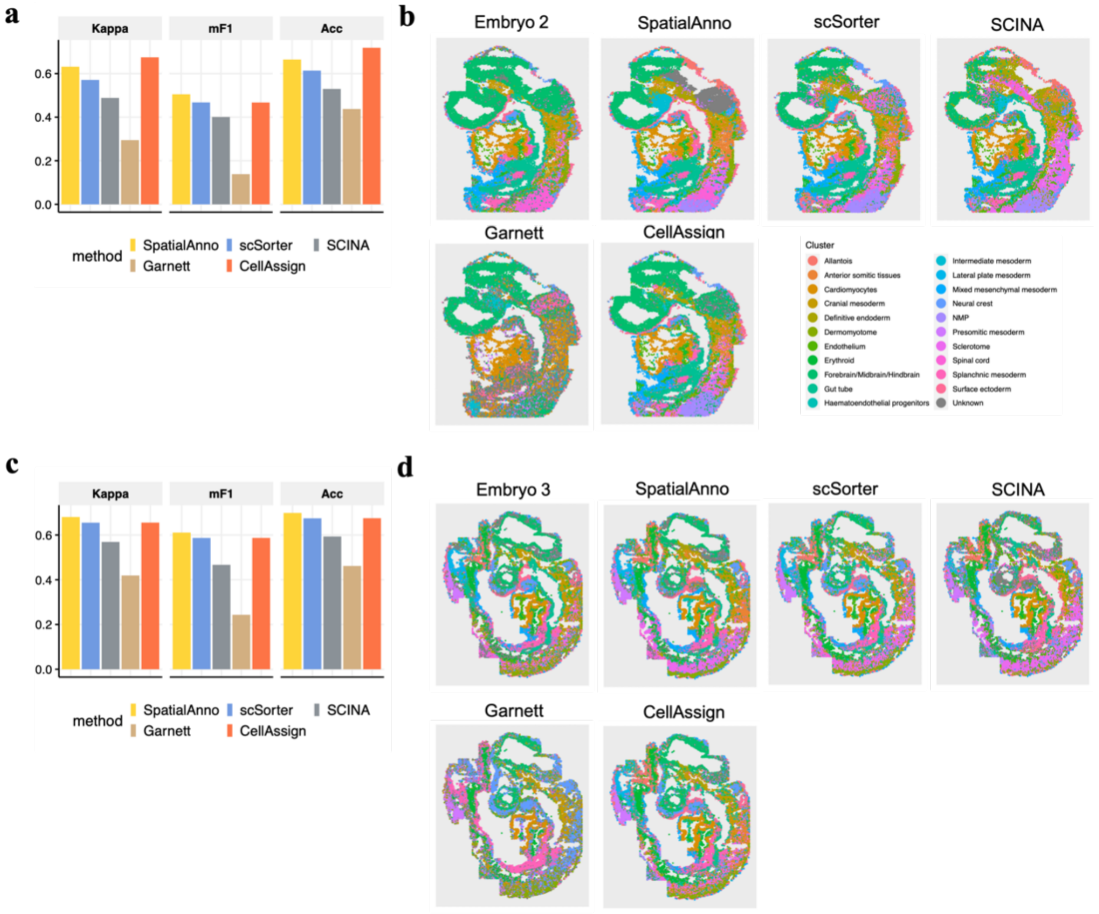

Supplementary Figure 28. Trajectory inference for mouse embryo 1 data

a Latent time trajectory generated by slingshot on low-dimensional embeddings of PCA. **b** Latent time trajectories generated by slingshot on low-dimensional DR-SC embeddings. **c** Heatmap of gene expression levels for the top 20 genes with significant expression changes with respect to the Slingshot pseudotime. Each column represents a spot that is mapped to this path and is ordered by its pseudotime value. Each row denotes the most significantly changed gene expression. **d** Boxplots of *Otx2* and *Sfrp1* expression counts in selected midbrain and hindbrain regions. **e** Spatial expression of *Otx2* and *Sfrp1* in brain region with corresponding virtual dissection (red line). For each location, only the gene with the higher expression value is plotted.

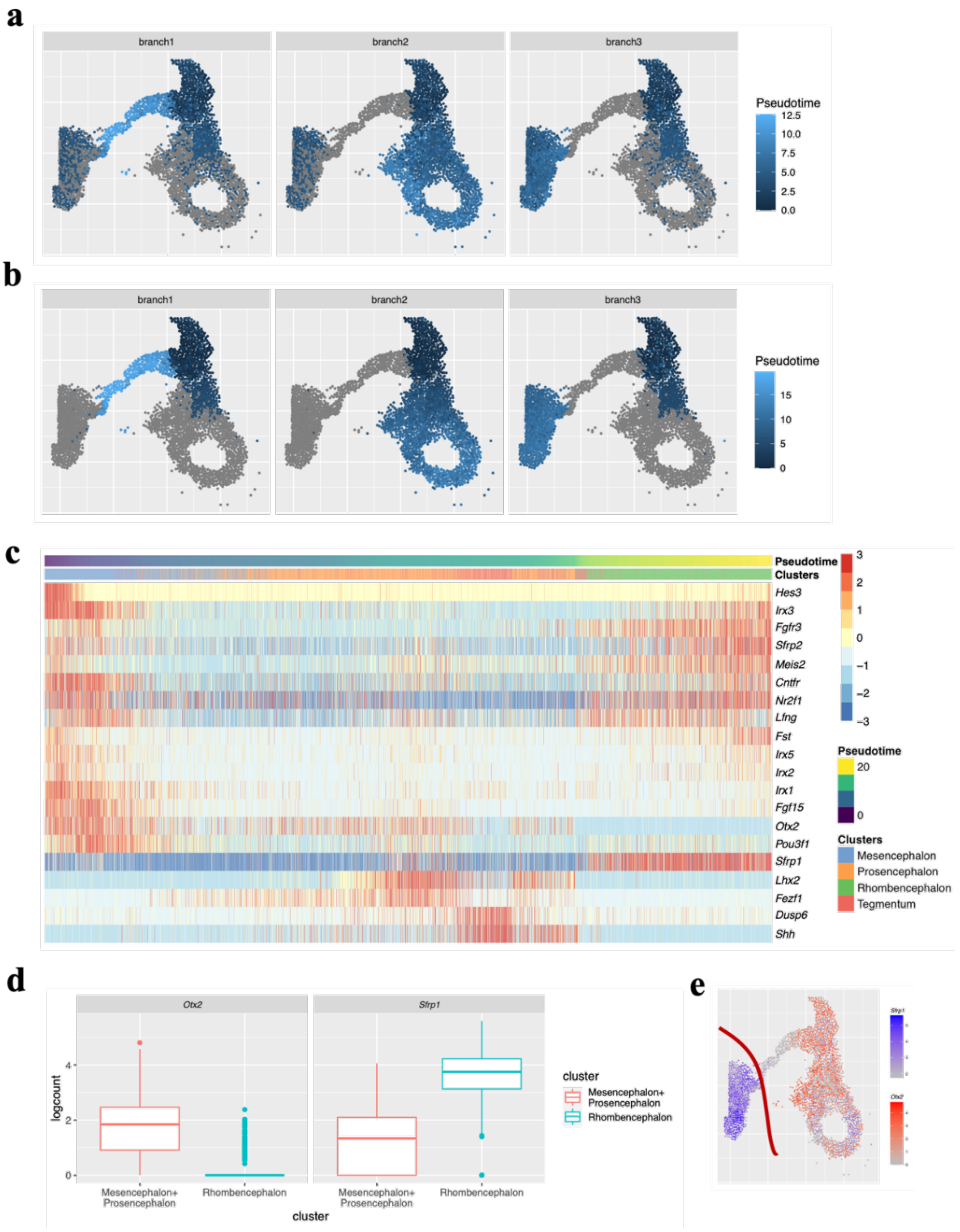

Supplementary Figure 29. Spatial annotation in real and simulated CyTOF data

a Boxplots of Kappa, mF1, and ACC showing the accuracy of different methods for cell type annotation across 522 imaging CyTOF data. We acquired a total of 548 images from a publication titled "Imaging mass cytometry and multiplatform genomics define the phenogenomic landscape of breast cancer”. To ensure the reliability of the analysis, we removed images with lower quality for downstream analysis. **b** Boxplots of Kappa, mF1, and ACC showing the accuracy of different methods for simulated data from the image named MB0633_1_76. The protein expression data were generated by the CyTOF data simulation tool Cytomulate.

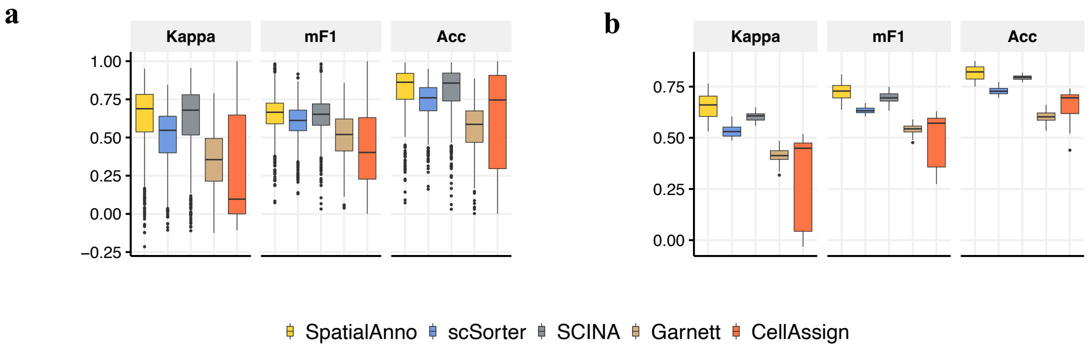

Supplementary Tables

Supplementary Table 1. Marker genes used in DLPFC dataset

Reference section	Type	Top 1-5 markers	Top 6-10 markers	Top 11-15 markers
**151507**	Layer 1	*Gfap,Fabp4,Snorc,Aqp4,Mt1g*	*Fabp7,Saa1,Cxcl14,Vim,Sparc*	*Agt,S100b,Cst3,Gja1,Mlc1*
	Layer 2	*Hpcal1,Enc1,Lamp5,Serpine2,Pcdh8*	*Hopx,Calb1,Atp2b1,Rasgrf2,Fkbp1a*	*Camk2n1,Sowaha,Hpca,Nnat,Itpka*
	Layer 3	*Cartpt,Enc1,Tespa1,Hopx,Hapln4*	*Gria4,Calb1,Nrgn,Pcdh8,Cyp46a1*	*Cbln4,Hs6st1,Itpka,Dbndd1,Yjefn3*
	Layer 4	*Nefh,Nefm,Nefl,Scn1b,Vamp1*	*Parm1,Pvalb,Map1b,Dclk1,Lgals1*	*Fabp3,Hbb,Slc30a3,Gpx3,Tuba1b*
	Layer 5	*Pcp4,Camk2d,Ipcef1,Tmsb10,Hbb*	*Scgb2a2,Smyd2,Efhd2,Tubb2a,Syt1*	*Scgb1d2,Pfkp,Syn2,Diras2,Sncg*
	Layer 6	*Krt17,Diras2,Efhd2,Nptx1,Tbr1*	*Scgb2a2,Scgb1d2,Tf,Scn3b,Mbp*	*Slc24a2,Syt1,Clstn2,Slc17a7,Slc35f1*
	WM	*Plp1,Mbp,Mobp,Cnp,Krt8*	*Tf,Mag,Cryab,S100a11,Krt18*	*Ppp1r14a,Septin4,Spp1,Bcas1,Cldnd1*
**151508**	Layer 1	*Gfap,Fabp4,Snorc,Aqp4,Saa1*	*Fabp7,Vim,Agt,Cxcl14,Sparc*	*Mt1g,S100b,Mlc1,Mt1m,Cst3*
	Layer 2	*Hpcal1,Lamp5,Enc1,Pcdh8,Cnr1*	*Serpine2,Hpca,Ncdn,Kcnip2,Gng2*	*Camk2n1,Itpka,Mfsd4a,Ppp3ca,Camkk1*
	Layer 3	*Enc1,Hopx,Rasgrf2,Lmo4,Ca10*	*Cyp46a1,Pcdh8,Tespa1,Rgs4,Nrgn*	*Hpcal1,Ptk2b,Hapln4,Hs6st3,C11orf87*
	Layer 4	*Nefm,Nefh,Hbb,Scrt1,Nefl*	*Vamp1,Scn1b,Pvalb,Dclk1,Gabra1*	*Lynx1,Map1b,Cplx1,Chga,Gars1*
	Layer 5	*Pcp4,Ipcef1,Clstn2,Tmsb10,Tubb2a*	*Stmn2,Scgb2a2,Syt1,Slc24a2,Pfkp*	*Snca,Neurod6,Tuba1b,Efhd2,Diras2*
	Layer 6	*Scgb2a2,Scgb1d2,Agr2,Iglc2,Krt8*	*S100a11,Mbp,Col1a2,Col1a1,Plp1*	*Mobp,Muc1,Diras2,Tf,Rps6*
	WM	*Mbp,Plp1,Mobp,Cnp,Tf*	*Bcas1,Cldnd1,Ppp1r14a,Cryab,Spp1*	*Mag,S100a11,Krt8,Paqr6,Lamp2*
**151509**	Layer 1	*Gfap,Aqp4,Saa1,Snorc,Mt1g*	*Vim,Fabp7,Agt,Cxcl14,Fabp4*	*Mt1m,Sparc,Cst3,Mgp,S100b*
	Layer 2	*Hpcal1,Enc1,Egr3,C1ql2,Nptx2*	*Pcdh8,Lamp5,Gng2,Ppp3ca,Cnr1*	*Psd3,Map2k1,Pcdh7,Ncdn,Serpine2*
	Layer 3	*Nefm,Cartpt,Lmo4,Gap43,Rgs4*	*Enc1,Necab1,Ywhah,Nrgn,Nefl*	*Chn1,Btbd8,C11orf87,Rbfox1,Calb1*
	Layer 4	*Nefh,Nefl,Nefm,Dclk1,Scn1b*	*Map1b,Tuba1b,Stmn2,Vamp1,Anxa6*	*Slc24a2,Chga,Tagln3,Spock1,Napb*
	Layer 5	*Pcp4,Igkc,S100a11,Agr2,Krt18*	*Efhd2,Krt8,Scgb1d2,Slc24a2,Tmsb10*	*Iglc2,Diras2,Syt1,Col1a2,Scgb2a2*
	Layer 6	*Col1a2,Krt8,S100a11,S100a10,Fn1*	*Col1a1,Col3a1,Muc1,Agr2,Krt18*	*Ccnd1,Krt19,Scgb1d2,Scgb2a2,Xbp1*
	WM	*Mbp,Plp1,Mobp,Tf,Cnp*	*Ppp1r14a,Cryab,Spp1,Cldnd1,Mag*	*Cldn11,Enpp2,Septin4,Rnase1,Mal*
**151510**	Layer 1	*Gfap,Aqp4,Mt1g,Snorc,Sparc*	*Cxcl14,Vim,Agt,Fabp7,Saa1*	*Mgp,Malat1,Cst3,Mt2a,S100b*
	Layer 2	*Hpcal1,Enc1,Pcdh8,Egr3,Lamp5*	*Serpine2,Pcdh7,Nptx2,Sst,Sowaha*	*Hopx,Ppp3ca,Ncdn,Baiap2,Gng2*
	Layer 3	*Cartpt,Nefm,Gap43,Nefl,Enc1*	*Hapln4,Lmo4,Sncg,Ywhah,Ldb2*	*Atp1b1,Chn1,Necab1,Oxr1,Stmn2*
	Layer 4	*Nefh,Nefl,Nefm,Dclk1,Tuba1b*	*Scn1b,Stmn2,Scn1a,Map1b,Ccni*	*Neurod6,Slc6a17,Vgf,Nrep,Ndrg4*
	Layer 5	*Pcp4,Ipcef1,Slc24a2,Gabra5,Tmsb10*	*Tubb2a,Efhd2,Syt1,Clstn2,Camk2d*	*Pfkp,Diras2,Slc35f1,Kcnab1,Tuba1b*
	Layer 6	*Krt8,Krt18,S100a10,Fn1,S100a11*	*Col1a2,Mbp,Col3a1,Krt19,Agr3*	*Col1a1,Plp1,Cldnd1,Slc24a2,Mobp*
	WM	*Mbp,Plp1,Mobp,Tf,Cnp*	*Mag,Ppp1r14a,Cldn11,Cryab,Cldnd1*	*Spp1,Bcas1,Enpp2,Rnase1,Mal*
**151673**	Layer 1	*Myl9,Malat1,Mgp,Tagln,Vim*	*Mt1g,Hla-a,Krt19,Cox6c,Atp8*	*Slc1a2,Cst3,Clu,Gfap,Nd5*
	Layer 2	*Hpcal1,Cxcl14,Lamp5,Camk2n1,Serpine2*	*Gnal,Calb2,Hopx,Itpka,Cnr1*	*Enc1,Rasgrf2,Gria2,Pcdh8,Rgs12*
	Layer 3	*Cartpt,Hopx,Enc1,Calb1,Nefm*	*Vstm2a,Lratd1,Gpx3,Hapln4,Cux2*	*Sncg,Nefl,Pcdh8,C11orf87,Ywhah*
	Layer 4	*Nefh,Nefm,Vamp1,Pvalb,Scn1b*	*Rorb,Nefl,Parm1,Ina,Vgf*	*Scn1a,Frmpd2,Dclk1,Gabrb2,Sncg*
	Layer 5	*Pcp4,Tmsb10,Pcp4l1,Smyd2,Gabra5*	*Ipcef1,Syt1,Diras2,Hs3st2,Tubb2a*	*Camk2d,Fam3c,Efhd2,Snap25,Clstn2*
	Layer 6	*Krt17,B3galt2,Scgb1d2,Diras2,Scgb2a2*	*Ifi27,Slc35f1,Tbr1,Krt19,Hla-b*	*Hs3st4,Scn3b,Mmd,Map2k1,Prkcb*
	WM	*Plp1,Mbp,Mobp,Cnp,Cryab*	*Tf,Mag,Ppp1r14a,Gfap,Cldn11*	*Ermn,Cldnd1,Spp1,Rnase1,Mog*
**151674**	Layer 1	*Malat1,Myl9,Mgp,Reln,Acta2*	*Cxcl14,Tagln,Cox6c,C11orf96,Csta*	*Bambi,Sparc,Vim,Igfbp7,Krt19*
	Layer 2	*Hpcal1,Serpine2,C1ql2,Cxcl14,Cnr1*	*Gnal,Sowaha,Enc1,Lamp5,Cux2*	*Camk2n1,Hopx,Sst,Calb2,Nptxr*
	Layer 3	*Cartpt,Enc1,Saa1,Hopx,Calb1*	*Fabp4,Ca10,Cux2,Pcdh8,Adcyap1*	*Nefm,Nsg2,Vstm2a,Cbln4,C11orf87*
	Layer 4	*Nefh,Nefm,Vamp1,Pvalb,Nefl*	*Scn1b,Parm1,Rorb,Syt2,Gpx3*	*Scn1a,Nsg1,Cntnap2,Tpbg,Sncg*

	Layer 5	*Pcp4,Hs3st2,Smyd2,Clstn2,Pcp4l1*	*Camk2d,Tubb2a,Ipcef1,Tmsb10,Nrep*	*Rorb,Nrn1,Vat1l,Fam3c,Syt1*
	Layer 6	*Cpb1,Scgb1d2,Scgb2a2,Krt17,B3galt2*	*Hla-b,Diras2,Krt19,Ifi27,Cox6c*	*Isg15,Bambi,Slc35f1,Tbr1,Scgb2a1*
	WM	*Plp1,Mbp,Mobp,Tf,Cnp*	*Mag,Cryab,Gfap,Ppp1r14a,Cldn11*	*Spp1,Ermn,Cldnd1,Bcas1,Carns1*
**151675**	Layer 1	*Azgp1,Myl9,Mgp,Tagln,Malat1*	*Vim,Cxcl14,Cst3,Slc1A2,Apoe*	*Clu,Camk2N1,Mtrnr2L8,Mt3,Nd14*
	Layer 2	*Hpcal1,Cxcl14,Serpine2,Saa1,Fabp4*	*Hopx,Cnr1,Itpka,Camk2N1,Sst*	*Enc1,Lamp5,Linc00507,Rgs12,Cartpt*
	Layer 3	*Cartpt,Hopx,Calb1,Fabp4,Enc1*	*Hs6St3,Saa1,Hapln4,Ca10,Pcdh8*	*Baiap3,Vstm2A,C11orf87,Nsg2,Tespa1*
	Layer 4	*Nefh,Nefm,Pvalb,Rorb,Nefl*	*Scn1B,Parm1,Sncg,Gpx3,Saa1*	*Vamp1,Tpbg,Lgals1,Nsg1,Cabp1*
	Layer 5	*Pcp4,Smyd2,Tmsb10,Pcp4L1,Tubb2A*	*Sncg,Syt1,Camk2D,Syn2,Clstn2*	*Cplx1,Snap25,Nrep,Etv1,Hs3St2*
	Layer 6	*Scgb1D2,Scgb2A2,Krt17,Krt19,B3Galt2*	*Hla-B,Diras2,Tbr1,Scn3B,Slc35F1*	*Rap1Gap2,Map2K1,Hs3St2,Slc17A7,Ly6H*
	WM	*Plp1,Mbp,Mobp,Tf,Cnp*	*Cryab,Gfap,Mag,Ppp1R14A,Ermn*	*Cldn11,Cldnd1,Spp1,Carns1,Mog*
**151676**	Layer 1	*Myl9,Tagln,Malat1,Cxcl14,Mt1G*	*Vim,Mgp,Fabp7,Sparc,Snorc*	*Aqp4,Atp8,Mt2A,Cst3,Mt1E*
	Layer 2	*Hpcal1,Cxcl14,Cnr1,Serpine2,Lamp5*	*Hopx,Itpka,Enc1,Sez6L,Camk2N1*	*Linc00507,Tespa1,Mt1G,Necab2,Fkbp1A*
	Layer 3	*Cartpt,Hopx,Enc1,Vstm2A,Calb1*	*Ca10,Cux2,Cbln4,Nefm,Saa1*	*Hapln4,Tespa1,Sncg,Linc01007,Adcyap1*
	Layer 4	*Nefh,Nefm,Pvalb,Saa1,Nefl*	*Scn1B,Vamp1,Sncg,Rorb,Frmpd2*	*Gpx3,Dclk1,Slc30A3,Parm1,Fabp4*
	Layer 5	*Pcp4,Tmsb10,Smyd2,Pcp4L1,Tubb2A*	*Hs3St2,Syn2,Snap25,Pfkp,Syt1*	*Diras2,Clstn2,Rorb,Ndrg4,Cdk14*
	Layer 6	*Scgb1D2,Scgb2A2,Cpb1,Krt17,B3Galt2*	*Diras2,Tff3,Krt19,Slc35F1,Tbr1*	*Hla-B,Bambi,Mmd,Scn3B,Slc17A7*
	WM	*Plp1,Mbp,Mobp,Tf,Cnp*	*Gfap,Cryab,Cldnd1,Mag,Ermn*	*Cldn11,Spp1,Mog,Ppp1R14A,Carns1*

Supplementary Table 2. Marker genes used in mouse OB dataset

Cell type	Markers
**Granule cells (GC)**	*Gria2, Meis2, Prkca, Penk*
**Periglomerular cells (PGC)**	*Nppa, Nrsn1, Nxph1, Th*
**Mitral and tufted cell (M/TC)**	*Cdhr1, Slc17a7, Olfm1, Reln*
**Olfactory sensory neurons (OSNs)**	*Gng13, S100a5, Omp, Fam213b*
**External plexiform layer interneuron (EPL-IN)**	*Kit, Thy1, Dner, Spock2*
**Endothelial**	*Ly6c1, Slco1a4, Ly6a, Cldn5*
**Mural**	*Cald1, Slco1a4, Ly6c1,Igfbp7*

Supplementary Table 3. Marker genes used in hippocampus dataset

Cell type	Markers
**Entorhinal cortex**	*Mef2c, Nrgn, Vsnl1, Snap25, Meg3*
**Ependymal**	*Dbi, Ccdc153, Rarres2, Tmem212, Nnat*
**CA3 Principal cells**	*Chgb, Hs3st4, Nptxr, Cpne4, Neurod6*
**Dentate hilum**	*Calb2, Pde1a, Rab3c, Satb1, Ajap1*
**Subiculum**	*Nov, Dcn, Gap43, Pou3f1, Pde1a*
**Interneuron**	*Gad2, Gad1, Cnr1, Slc6a1, Nrxn3*
**CA1 Principal cells**	*Wfs1, Fibcd1, Atp2b1, Itpka, Ppp3ca*
**Oligodendrocyte**	*Plp1, Ptgds, Mbp, Mal, Mag*
**Astrocyte**	*Apoe, Cst3, Aldoc, Mt1, Clu*
**Endothelial stalk**	*Ly6c1, Bsg, Flt1, Itm2a, Ly6a*
**Endothelial tip**	*Ptgds, Apod, Igf2, Igfbp2, Col1a2*
**Polydendrocyte_2**	*Mbp, Sirt2, Plp1, Cnp, Mag*
**Polydendrocyte_1**	*Olig1, Cacng4, Ptprz1, Marcks, Lhfpl3*
**Mural**	*Acta2, Tpm1, Tpm2, Crip1, Tagln*
**Dentate Principal cells**	*C1ql2, Ppp3ca, Fam163b, Olfm1, Ncdn*
**Neurogenesis (SGZ)**	*Sox4, Tubb5, Sox11, Rps5, Rps9*
**Hb neurons**	*Rora, Slc17a7*
**Choroid**	*Ttr, Enpp2,1500015o10rik, Prlr, Igfbp2*
**Cajal_Retzius**	*Pcp4, Ndnf, Ramp1, Gap43, Reln*
**Neuron.Slc17a6**	*Camk2n1, Nrgn, Cck, Tshz2, Arpp21*
**Microglia_Macrophages**	*Hexb, Cst3, Ctss, Cx3cr1, C1qb*

Supplementary Table 4. Marker genes used in embryo dataset

Cell type	Markers
**Lateral plate mesoderm**	*Pitx1, Cdx4, Evx1, Cdx2, Hand2, Hoxa9, Hoxc6, Hoxc9*
**Erythroid**	*Slc4a1, Alas2, Klf1, Gata1, Epor, Acp5, Hemgn, Smim1*
**Allantois**	*Tbx4, Pitx1, Hand1, Wnt2, Col1a1, Hand2, Msx1, Tbx3*
**Gut tube**	*Krt18, Foxa1, Cldn4, Shh, Cdh1, Myh9, Cpm, Itga3*
**Endothelium**	*Cdh5, Plvap, Eng, Cd34, Cldn5, Kdr, Sox18, Pecam1*
**Hematoendothelial progenitors**	*Cldn5, Sox18, Cdh5, Plvap, Cd34, Eng, Kdr, Sox7*
**Intermediate mesoderm**	*Lef1, Pitx1, Evx1, Tbx3, Tbx4, Bmp4, Cdx2, Cdx4*
**Mixed mesenchymal mesoderm**	*Hand1, Col1a1, Tmem108, Ahnak, Dlk1, Gata6, Postn, Smoc2*
**Spinal cord**	*Hoxb9, Hoxd4, Sox2, Hoxb8, Foxb1, Hoxc6, Hoxc8, Foxa2*
**Neural crest**	*Sox10, Tfap2b, Tfap2a, Nr2f1, Prrx1, Snai1, Msx1, Alx1*
**Splanchnic mesoderm**	*Foxf1, Osr1, Hoxb1, Isl1, Gata5, Tbx5, Kcng1, Gata4*
**Forebrain/Midbrain/Hindbrain**	*En1, Fgf15, Otx2, Sox2, Pou3f1, Lfng, Lhx2, Hes3*
**Cranial mesoderm**	*Tbx1, Col26a1, Foxc2, Marcks, Tmem119, Cxcl12, Col1a2, Fst*
**Surface ectoderm**	*Pdgfa, Tfap2a, Cdh1, Epcam, Cldn4, Itga3, Krt18, Gjb3*
**Definitive endoderm**	*Shh, Foxa1, Foxa2, Cdh2, T, Cldn4, Irx1, Irx3*
**NMP**	*Hoxb9, Hoxc8, Hoxd4, Sox2, Hoxb4, Hoxc6, Foxb1, Hoxb8*
**Anterior somitic tissues**	*Meox1, Foxc2, Col26a1, Cxcl12, Snai1, Marcks, Aldh1a2, Fst*
**Presomitic mesoderm**	*Meox1, Dll3, Foxc2, Dll1, Lef1, Cer1, Notch1, Mesp2*
**Dermomyotome**	*Meox1, Aldh1a2, Six1, Hoxb3, Col26a1, Foxc2, Hoxb4, Fst*
**Cardiomyocytes**	*Popdc2, Atp1b1, Tagln, Ttn, Smarcd3, Gata5, Tbx5, Hcn4*
**Sclerotome**	*Meox1, Aldh1a2, Foxc2, Col26a1, Cxcl12, Marcks, Pdgfra, Snai1*

Supplementary Table 5. List of 12 DLPFC sections

Section ID	Species	Protocol	Year	No. genes	No. spots
**151507**	Human	10x Visium	2021	33538	4226
**151508**	Human	10x Visium	2021	33538	4384
**151509**	Human	10x Visium	2021	33538	4789
**151510**	Human	10x Visium	2021	33538	4634
**151669**	Human	10x Visium	2021	33538	3661
**151670**	Human	10x Visium	2021	33538	3498
**151671**	Human	10x Visium	2021	33538	4110
**151672**	Human	10x Visium	2021	33538	4015
**151673**	Human	10x Visium	2021	33538	3639
**151674**	Human	10x Visium	2021	33538	3673
**151675**	Human	10x Visium	2021	33538	3592
**151676**	Human	10x Visium	2021	33538	3460

Supplementary Table 6. List of 12 mouse olfactory bulb (MOB) sections

Dataset	Species	Protocol	Year	No. genes	No. locations
**MOB Rep1**	Mouse	ST	2016	16573	267
**MOB Rep2**	Mouse	ST	2016	15981	280
**MOB Rep3**	Mouse	ST	2016	16014	269
**MOB Rep4**	Mouse	ST	2016	15941	264
**MOB Rep5**	Mouse	ST	2016	15290	267
**MOB Rep6**	Mouse	ST	2016	16251	242
**MOB Rep7**	Mouse	ST	2016	16675	231
**MOB Rep8**	Mouse	ST	2016	15288	234
**MOB Rep9**	Mouse	ST	2016	15284	237
**MOB Rep10**	Mouse	ST	2016	16416	281
**MOB Rep11**	Mouse	ST	2016	16218	262
**MOB Rep12**	Mouse	ST	2016	16034	282

Supplementary Table 7. Features of two mouse hippocampus sections

Section	Species	Protocol	Year	No. genes	No. locations
**1**	Mouse	Slide-seq	2021	22457	34199
**2**	Mouse	Slide-seqV2	2021	23264	53208

Supplementary Table 8. Features of three mouse embryo sections

Section	Specie	Protocol	Year	No. genes	No. locations
**1**	Mouse	seqFISH	2021	351	19451
**2**	Mouse	seqFISH	2021	351	14891
**3**	Mouse	seqFISH	2021	351	23194

Supplementary Table 9. Computation time for four real data applications

Computing time in seconds was recorded using a single thread on a 2.1 GHz Intel Xeon Gold 6230 CPU with 16 GB memory

Method	DLPFC data (average time for 12 sections)	Mouse OB data (average time for 12 sections)	Slide-seq V2 data	seqFISH data (average time for 3 sections)
**SpatialAnno**	137.5	2.3	5667.6	6272.8
**scSorter**	131.5	7.5	3262.7	1512.5
**SCINA**	3.8	0.1	44.9	23.3
**Garnett**	127.9	168.9	193.1	59.5
**CellAssign**	241.7	15.8	30608.6	16209.7
